# Single‐Cell RNA Sequencing Reveals the Heterogeneity in Differentiation Trajectory and Tumor Microenvironment Leading to More Aggressive Phenotypes of Papillary Thyroid Cancer in Children and Young Adult Patients

**DOI:** 10.1002/advs.202417672

**Published:** 2025-07-28

**Authors:** Kai Guo, Lingyi Yang, Zirui Huang, Kai Qian, Yuan Shi, Mengjia Fei, Yuan Feng, Zixian Chen, Ming Qiu, Linglin Tang, Anqi Li, Zhibao Lv, Jiangbin Liu, Zhou Chen, Kuiran Dong, Zhuoying Wang

**Affiliations:** ^1^ Department of Head and Neck Surgery Renji Hospital School of Medicine Shanghai Jiaotong University Shanghai 200001 China; ^2^ Department of Nuclear Medicine Renji Hospital School of Medicine Shanghai Jiaotong University Shanghai 200001 China; ^3^ Department of Pathology Ruijin Hospital School of Medicine Shanghai Jiaotong University Shanghai 200001 China; ^4^ Department of General Surgery Children's Hospital of Shanghai Shanghai Jiao Tong University Shanghai 200333 China; ^5^ Department of Pediatric Surgery Children's Hospital of Fudan University Shanghai 201102 China

**Keywords:** ⁶⁸Ga‐FAPI‐PET, papillary thyroid cancer, single‐cell RNA sequencing, thyrocyte differentiation trajectory, tumor microenvironment

## Abstract

The tumor ecosystem heterogeneity of papillary thyroid carcinoma (PTC) is poorly characterized in children and young adult patients (CAYA‐PTC). In this study, single‐cell RNA sequencing is used to profile transcriptomes from the paratumor and tumor tissues of 11 patients. Compared to adult, CD4T_Tfh and CD8T_Tex cells are significantly more prevalent in CAYA‐PTC patients. Three phenotypes are identified within the thyrocytes through differentiation trajectory analysis, including normal, BRAF‐like, and Fusion‐like. Notably, the data reveal that CAYA‐PTC patients lack the “mild‐state (BRAF‐like)” malignant thyrocyte population. This variation in differentiation states indicates that PTC cells in CAYA patients rapidly develop into invasive and metastatic forms, whereas in adult patients, this progression occurs gradually over a longer period. Additionally, extracellular matrix cancer‐associated fibroblasts (emCAFs_LAMP5) interact with endothelial cells and thyrocytes, promoting tumor angiogenesis and metastasis more prominently in CAYA patients. Fibroblast activation protein (FAP) expression is high in emCAF_LAMP5 and positively correlated with LAMP5 in CAYA‐PTC tissues. Consequently, 68Ga‐FAPI‐PET emerges as a promising diagnostic method for CAYA patients who are not effectively diagnosed by traditional 18F‐FDG‐PET. Collectively, the findings provide insight into the CAYA‐PTC ecosystem that suggests distinct diagnostic, prognostic, and therapeutic implications compared to adults.

## Graphical abstract

scRNA sequencing reveals distinct tumor ecosystem features in CAYA‐PTC, marked by enriched CD4T_Tfh, CD8T_Tex cells, and rapid malignant thyrocyte differentiation (absent of mild‐state BRAF‐like population) toward invasive states. Prominent emCAF_LAMP5 populations interact with endothelial cells and thyrocytes facilitate angiogenesis and metastasis, highlighting ^68^Ga‐FAPI‐PET as a potential superior diagnostic tool over traditional ^18^F‐FDG‐PET for CAYA‐PTC.

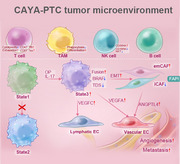

## 1 Introduction

Papillary thyroid cancer (PTC), constituting the predominant histological subtype among thyroid malignancies, is characterized by its heterogeneous clinical behavior and complex molecular landscape.^[^ ^1^ ^]^ In children and young adults (CAYA‐PTC), the clinical presentation and tumor behavior are significantly more aggressive than in adult patients (Adult‐PTC), manifesting as larger tumor sizes, earlier capsule involvement, and more extensive lymph node metastases.^[^ ^2^, ^3^ ^]^ These differences warrant thorough investigation and discussion. While substantial progress has been made in deciphering the genomic alterations within PTC cells, the tumor microenvironment (TME) remains a pivotal yet enigmatic player in disease progression. The intricate interplay between cancer cells and the cellular components of the TME, including fibroblasts, immune cells, and vasculature, plays a crucial role in shaping the phenotypic diversity and therapeutic response of thyroid cancer.^[^ ^4^, ^5^, ^6^ ^]^ Some studies have reported a correlation between clinical outcomes and tumor‐infiltrating immune cells in thyroid cancer patients. For instance, BRAFV600E promotes thyroid cancer development by increasing the penetrance of myeloid‐derived suppressor cells, which exhibit strong immunosuppressive potential and are associated with a poor prognosis.^[^ ^7^ ^]^ Furthermore, distinct transcriptional and functional changes in myeloid cells arise before their infiltration into the tumor and are initiated in bone marrow, suggesting an active role in forming the tumor immune microenvironment.^[^ ^8^ ^]^ M2‐macrophages downregulate *METTL3* in the thyroid cancer cells, leading to the stabilization and increase of *CD70*, which results in a higher proportion of regulatory T cells (Tregs) and terminally exhausted T cells.^[^ ^9^ ^]^ Additionally, the infiltration of *CD3*(−)*CD16*(−)*CD56*(bright) immunoregulatory natural killer (NK) cells inversely correlates with advanced stages of PTC, highlighting their vital role in thyroid cancer immunosurveillance escape.^[^ ^10^ ^]^ Thus, characterizing both tumor cells and immune cells is crucial to understanding their properties and interactions in PTC.

Traditional bulk sequencing methods, while informative, are limited in their ability to reveal the heterogeneity within the TME. The emergence of single‐cell sequencing (scRNA) technologies provides a revolutionary tool to dissect the cellular and molecular landscape of PTC with unprecedented resolution. This study leverages scRNA sequencing to unravel the complexity of the PTC microenvironment, highlighting the diverse cellular populations and their dynamic interactions. Our focus is on the tumor microenvironment discrepancy within PTC between CAYA and adult‐PTC patients. By applying scRNA sequencing to immune cell and thyrocyte populations, we aim to identify distinct subsets of T cells, B cells, NK cells, myeloid cells, endothelial cells, and thyrocytes, thus providing insight into the immune landscape and exploring potential immune evasion mechanisms. Furthermore, our investigation extends to the stromal compartment, exploring the contributions of cancer‐associated fibroblasts and endothelial cells to tumor progression and angiogenesis.

By employing this comprehensive approach, we aim to uncover novel molecular players and regulatory networks within the PTC microenvironment. Identifying these key elements holds the potential to enhance targeted diagnostic and therapeutic strategies aimed at modulating the TME, ultimately improving outcomes for CAYA‐PTC patients.

## 2 Results

### 2.1 Generation of a Single‐Cell Atlas for PTC

**Figure 1:**
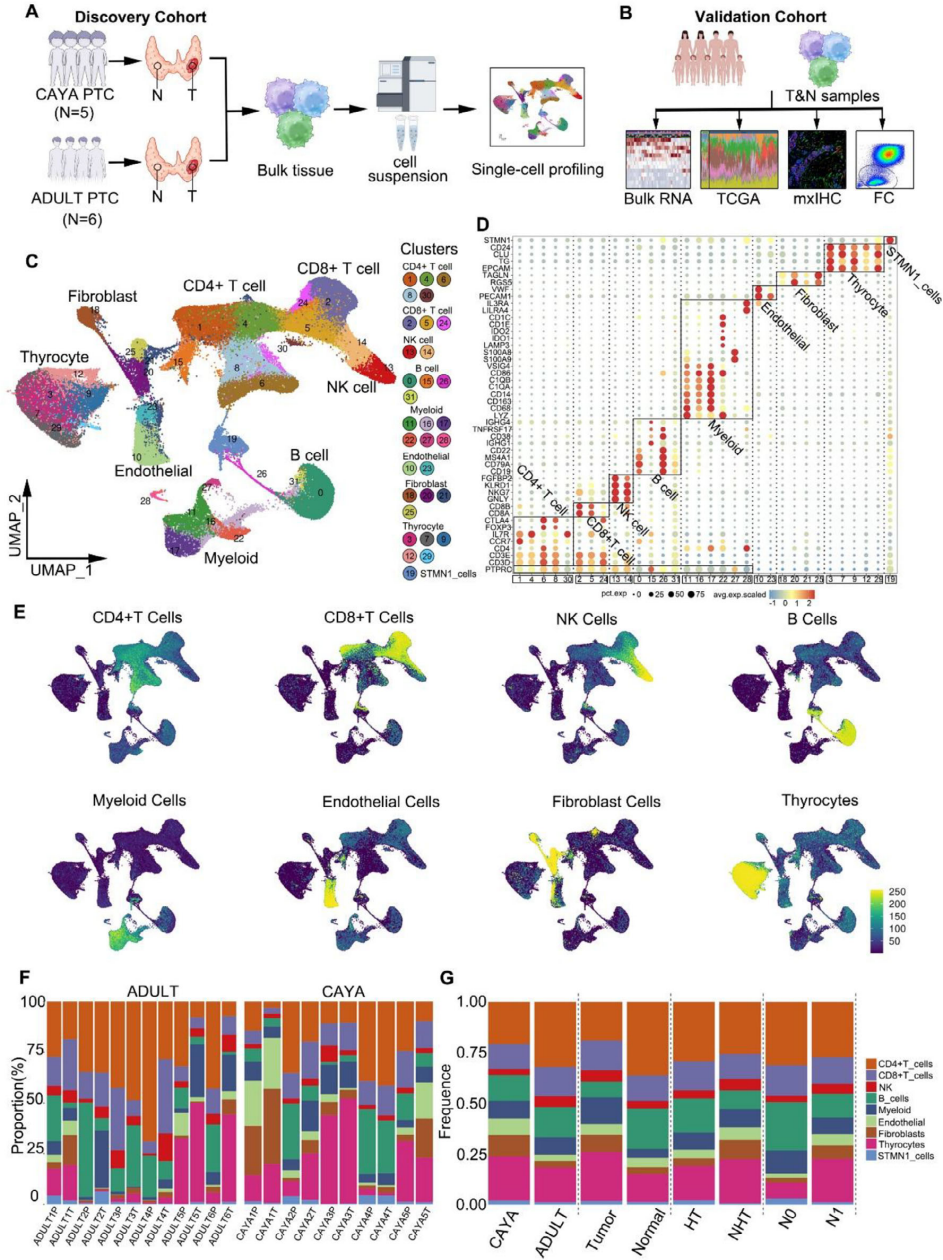
The single‐cell atlas of PTC. A,B) Schematic representation of the experimental strategy. The discovery cohort is shown in (A), and the validation cohorts in (B). The numbers (*n*) of patients in each subgroup are provided in the figure. C) Uniform manifold approximation and projection (UMAP) plot, showing the annotation and color codes for cell types in the PTC ecosystem. D) Bubble plot showing the expression of marker genes in the indicated cell types. The bottom bars label the clusters corresponding to specific cell types, and the number in rectangular box correspond to the cluster number in (C). E) Expression of genes correlated with immune, stromal, epithelial cell (thyrocytes) types by Ucell. F) Fraction of each subtype from different samples. G) Fraction of each subtype from different groups (CAYA: children and young adults; N: normal tissue; T: tumor tissue; FC: flow cytometry; HT: cowith‐Hashimoto's thyroiditis; NHT: with no Hashimoto's thyroiditis).

To understand the complexity of the tumor ecosystem and dynamic interactions between malignant, stromal, and immune cells in PTC, we designed our study by discovery and validation cohorts. Additional public data including five paired adult samples were integrated in analysis^[^ ^6^ ^]^ (Table S1, Supporting Information). All tumor samples were collected at diagnosis (**Figure** **1**A). Whole transcriptome RNA sequencing was also conducted on 50 patients (validation cohort 1; 40 CAYA and 10 adult cases, see Excel S1, Supporting Information), as well as on an additional 550 samples from The Cancer Genome Atlas (TCGA) (validation cohort 2; 493 tumor and 57 normal tissues), to validate the subclustering based on scRNA gene expression and to assess the ecological heterogeneity of PTC. Moreover, multiplexed immunohistochemistry (mxIHC) validation was conducted on 78 paired CAYA and 80 paired adult tissues, each with paired tumor and normal samples (validation cohort 3, see Excel S2, Supporting Information). The contents of T cells, B cells, NK cells, and myeloid cells in tumor tissue and paratumor tissue from 10 CAYA and 10 adults with PTC (validation cohort 4, see Excel S3, Supporting Information) were detected by flow cytometry to confirm differences observed in the discovery cohort (Figure 1B).

Preliminary cell clustering was performed using a shared nearest neighbor modularity optimization‐based clustering algorithm. After removing the batch effect, the major cell type and lineage of each cluster were determined based on the expression of canonical markers (Figure S1C, Supporting Information). In total, we identified 40 053 CD4+ T cells (27.92%), 19 534 CD8+ T cells (13.61%), 6547 NK cells (4.56%); 20 159 B cells (14.05%), 12 439 myeloid cells (8.67%), 7133 endothelial cells (4.97%), 8338 cancer‐associated fibroblasts (CAFs) (5.81%), 26 933 thyrocyte cells (18.77%), and 2341 STMN1 cells (1.63%) (Figure 1C). Furthermore, based on top differentially expressed genes, we identified unique markers for each of the 32 major clusters (Figure 1D) and used the machine‐learning algorithm “Garnett” to assign signatures to each cluster (Figure S1A,B, Supporting Information). To further verify the reliability of clustering, we projected key gene scores onto the uniform manifold approximation and projection (UMAP) using Ucell, and the results were consistent with our clustering (Figure 1E; Table S2, Supporting Information). This allowed us to develop a cluster‐agnostic approach for cell‐type annotation. For each individual sample, the proportion of thyrocyte cell types was highly variable, with CD4+ T cells being the most dominant cell type within the TME of PTC, followed by B cells, CD8+ T cells, myeloid cells, fibroblasts, NK cells, and endothelial cells (Figure 1F). We also discovered distinct fibroblast and myeloid cells, as well as B cells and CD4+ T cells, in the tumor and adjacent normal tissue (Figure 1G; Figure S1D,E and Excel S4, Supporting Information). Notably, cancer‐associated fibroblast cells and endothelial cells were specifically enriched in CAYA patients, while CD4+/CD8+ T cells, NK cells, and B cells were predominantly present in adult patients (Figure 1G; Figure S1D,E, Supporting Information). Overall, our comprehensive analysis of 143 477 single cells from normal and tumor tissue of the human thyroid provides an unprecedented opportunity to compare this atlas across subgroups.

### 2.2 The Heterogeneity of CD4+ T Cells in Developmental State and Clonalities in PTC

**Figure 2:**
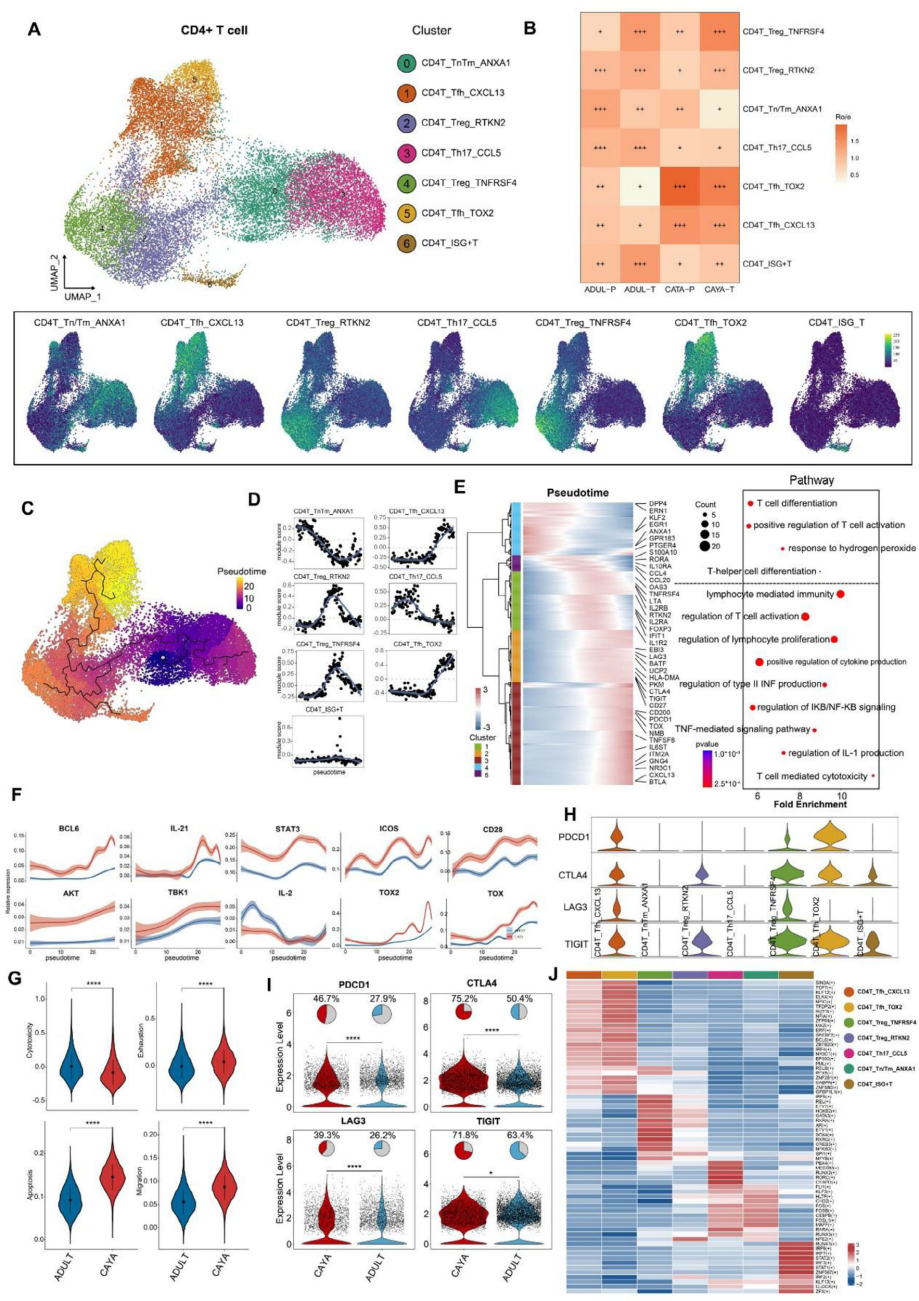
The heterogeneity, development, and clonality of CD4+ T cells in PTC. A) UMAP (upper) of CD4+ T cells and Ucell mapping of key markers (lower) for CD4+ T cell subclusters. Naive/memory T(Tn/m, c0), Follicular helper T(Tfh, c1, 5), Treg (c2, 4), Helper T (Th17, c3), ISG+T (c6). Color gradients in the right upper legend reflect the magnitude of signature enrichment, with higher scores indicating stronger expression. B) Tissue preference of each CD4+ T cell cluster estimated by the ratio of observed to expected cell numbers (Ro/e). C) Pseudotime trajectory analysis of all CD4+ T cells with high variable genes. The inset UMAP plot shows each cell with a pseudotime score from dark blue to yellow, indicating early to terminal states, respectively. D) 2D plots showing the expression scores for genes related to each cluster along with the pseudotime. E) Heatmap showing the dynamic changes in gene expression along the pseudotime. The distribution of CD4+ T subtypes during the transition (divided into five phases), along with the pseudotime. F) Shaded line plot indicating the expression levels of the selected Tfh‐related genes along the pseudotime in CAYA and adult patients. G) Differences in six functional scores between CAYA and adult groups. *P < 0.05, **P < 0.01, ***P < 0.001, ****P < 0.0001; two‐sided *t*‐test. H) Violin plot indicating the expression of exhaustion related genes (*PDCD1*, *CTLA4*, *LAG3*, *TIGIT*) in seven subclusters of CD4+ T cells. I) Violin plot indicating the expression of exhaustion related genes (*PDCD1*, *CTLA4*, *LAG3*, *TIGIT*) in CD4+ T cells in CAYA (red) and adult (blue) patients. *P < 0.05, **P < 0.01, ***P < 0.001, ****P < 0.0001; two‐sided *t*‐test. J) Heatmaps of the activation scores of each CD4+ T cell cluster for expression regulated by transcription factors (TFs). T cell clusters are indicated on top, and the scores were estimated using SCENIC analysis.

CD4+ T cells (*n* = 40053) represented the most prevalent cell type in PTC. Reclustering revealed seven distinct clusters: Tn/Tm_ANXA1, Tfh_CXCL13, Treg_RTKN2, Th17_CCL5, Treg_TNFRSF4, Tfh_TOX2, and CD4T_ISG+T (**Figure** **2**A), each exhibiting different tissue preference patterns (Figure 2B). The Tfh_CXCL13 and Tfh_TOX2 were predominantly enriched in CAYA tissues, suggesting a more immunosuppressive status compared to adult patients (Figures 2B and 6A,B). Specifically, Treg_TNFRSF4 cells showed increased expression of *TNFRSF4*, *TNFRSF9*, and *LYAN*, similar to previously reported immunosuppressive TNFRSF9+ Tregs^[^ ^11^, ^12^ ^]^ and Treg LAYN,^[^ ^13^, ^14^ ^]^ and were present in higher proportions in both adult and CAYA tumor sites (Figures 2B and 6A–C). In contrast, Treg_RTKN2 was highly expressed only in CAYA tumor tissues compared with paratumor tissues (Figure 2B). In CAYA‐PTC samples, the proportion of CD4+ T cells among CD45+ T lymphocytes decreased, while the proportion of CD25+CD127‐Treg cells among CD4+ T cells increased (14.5 ± 3.9%), which was significantly higher than in adults (9.8 ± 3.5%, *P* < 0.05) (Figure S2A–C and Table S3, Supporting Information).

We next explored the dynamic immune states and cell transitions in PTC‐infiltrated CD4+ T cells by inferring state trajectories using Monocle 3.^[^ ^15^ ^]^ The starting states were set as the branch containing most naive T cell subclusters. Three major trajectories were identified (Figure 2C). T cells developed from Tn/Tm_ANXA1 to Th17_CCL5 (trajectory 1), Treg and ISG+T (trajectory 2), Tfh (trajectory 3). Gene set scores over pseudotime clearly show the score peaks consistent with predicted theoretical analysis (Figure 2D). We next investigated the transcriptional changes associated with transitional states and observed that CD4+ T cells could be categorized into five phases (Figure 2E). Tn/Tm_ANXA1 and Th17_CCL5 cells were predominantly enriched in state 4 and 5, characterized by upregulated expression of *KLF2*, *EGR1*, *ANXA1*, and *GPR183*, suggesting these cells had the lowest cytotoxic capacities. Cells in states 1, 2, and 3 demonstrated the highest expression levels of *FOXP3*, *CTLA4*, *TIGIT*, *OAS3*, *IFIT1*, *TOX*, *NR3C1*, and *CXCL13*, matching the phenotypes of Treg cells, ISG+ T cells, and Tfh cells (Figures 2E).^[^ ^16^, ^17^, ^18^ ^]^ Pathway analysis suggested that cells were involved in the regulation of T cell activation, confirming the dynamic transition states toward exhausted phenotype. Cells in the end of trajectory demonstrated the highest expression levels of *BCL6*, *IL‐21*, *STAT3*, *ICOS*, *CD28*, *AKT*, and *TBK1*, which are positively corelated with *TOX* and *TOX2*, matching the phenotypes of Tfh cells.^[^ ^19^ ^]^ Importantly, the expression of these Tfh‐related genes was higher in CAYA than that in adult, indicating a stronger suppressive role of Tfh cells in CAYA patients (Figure 2F).

We further compared the expression levels of cytotoxicity, exhaustion, apoptosis, and migration scores between CAYA and adult groups. The results indicated that cytotoxicity score was higher in adult patients (0.01 ± 0.18 vs −0.08 ± 0.19), while the exhaustion (−0.003 ± 0.128 vs 0.046 ± 0.127), apoptosis (0.092 ± 0.03 vs 0.145 ± 0.029), and migration (0.055 ± 0.028 vs 0.087 ± 0.026) scores were higher in CAYA group (Figure 2G). Here, although statistically significant, the observed difference in exhaustion scores between CAYA and adult cohorts represented a small effect size, suggesting the biological relevance of this numerical difference may be limited. We also calculated the immune checkpoint scores for CD4+ T cell clusters, revealing lower average expression of immune checkpoint scores in adult patients in Tfh_CXCL13, Treg_RTKN2, Treg_TNFRSF4, and Tfh_TOX2 subtypes (Figure 2H,I). Subsequently, SCENIC analysis also revealed a potential regulatory network of TFs across subtypes in PTC (Figure 2I). For example, *BCL6* regulate most of the key targets required for Tfh cell formation.^[^ ^20^ ^]^ Th17 cell gene expression is regulated by the transcription factor retinoid orphan nuclear receptor.^[^ ^21^, ^22^ ^]^

Collectively, through the integrated analysis of single‐cell transcriptome data, we identified multiple CD4+ T cell populations with distinct distribution patterns and revealed unique dynamics of T cells in CAYA and adult. Most importantly, our findings indicate that CAYA‐enriched Treg/Tfh cells play a potential vital role in shaping the TME during T cell infiltration, ultimately promoting tumor immunosuppression.

### 2.3 CD8+ T Cell Lineages Reveal Evolutionary Trajectories and the Influence of Exhaustion on CAYA Patients

**Figure 3:**
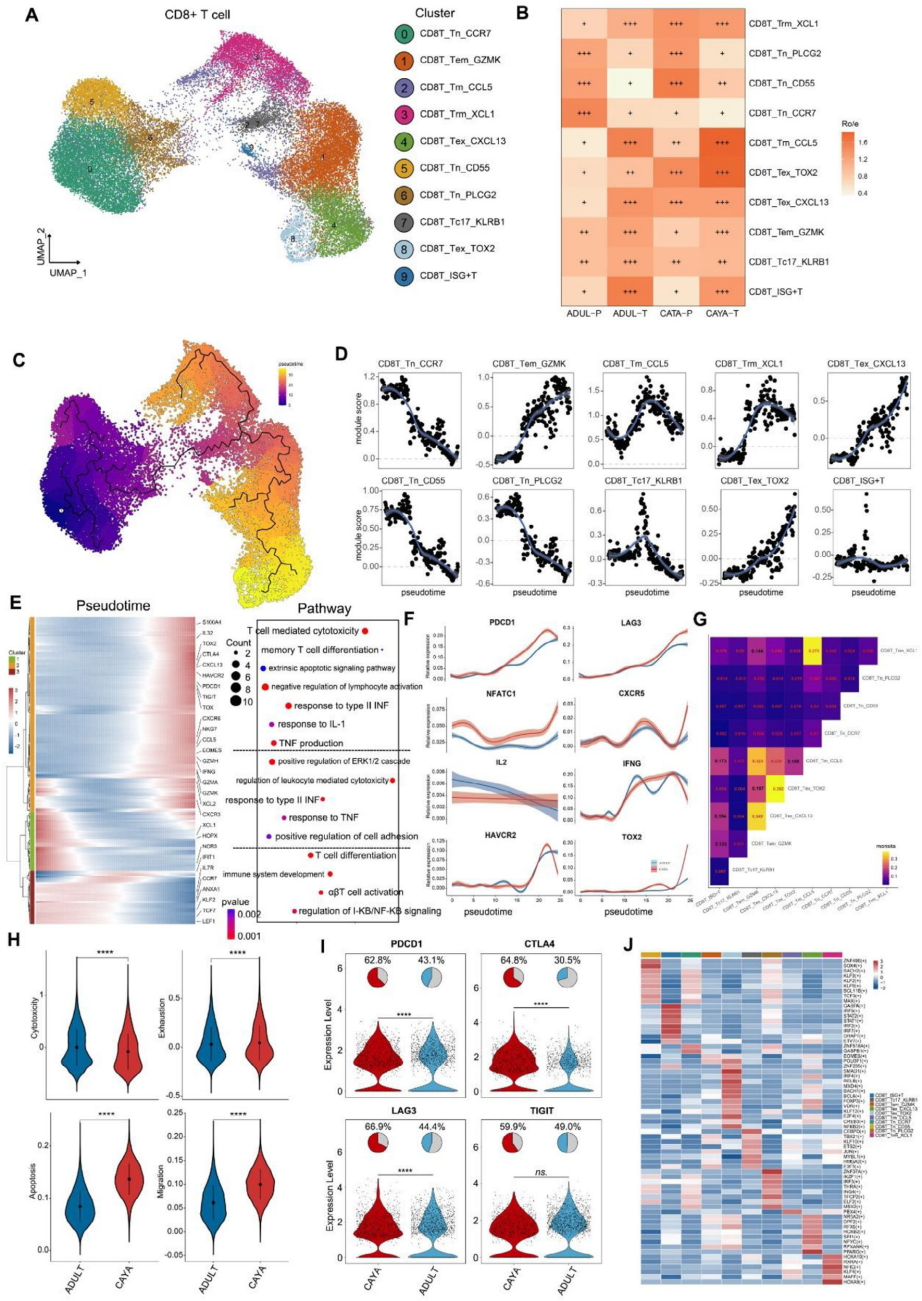
The heterogeneity, development, and clonality of CD8+ T cells in PTC. A) UMAP of CD8+ T cells and Ucell mapping of key markers for CD8+ T cell subclusters. Naïve T (Tn, c0,5,6), effector memory T (Tem, c1), memory T (Tm, c2), tissue resident memory T (Trm, c3), exhausted T (Tex, c4, 8), cytotoxic T (Tc17, c7), and ISG+T (c9). B) Tissue preference of each CD8+ T cell cluster estimated by the ratio of observed to expected cell numbers (Ro/e). C) Pseudotime trajectory analysis of all CD8+ T cells with high variable genes. The inset UMAP plot shows each cell with a pseudotime score from dark blue to yellow, indicating early to terminal states, respectively. D) 2D plots showing the expression scores for genes related to each cluster along with the pseudotime. E) Heatmap showing the dynamic changes in gene expression along the pseudotime. The distribution of CD8+ T subtypes during the transition (divided into three phases), along with the pseudotime. F) Shaded line plot indicating the expression levels of the selected Tex‐related genes along the pseudotime in CAYA and adult patients. G) Heatmap showing the developmental transition potential between CD8+ T cells quantified by pairwise STARTRAC. H) Differences in six functional scores between CAYA and Adults groups. *P < 0.05, **P < 0.01, ***P < 0.001, ****P < 0.0001; two‐sided *t*‐test. I) The expression violin plots showing the immune checkpoint genes by dividing into CAYA and adult groups. *P < 0.05, **P < 0.01, ***P < 0.001, ****P < 0.0001; two‐sided *t*‐test. J) Heatmaps of the activation scores of each CD8+ T cell cluster for expression regulated by transcription factors (TFs).

Subclustering of the CD8+ T cell compartment resulted in the identification of ten clusters (**Figure** **3**A). These clusters represent different T cell functional states, including naïve (c0, 5, 6), memory (c1, 2, 3), cytotoxic T (c7), exhaustion (c4, 8), and ISG+T (c9) cells based on the expression of canonical marker genes (Figure S3A, Supporting Information). Overall, the proportion of CD8+ T cells in PTC tumor tissues of children (13.8 ± 3.7%) was significantly lower than that of adults (22.4 ± 5.1%, *P* < 0.05) (Figure S2D and Table S3, Supporting Information). Of which, two exhausted T cell clusters were identified based on elevated expression of genes including *TOX*, *TOX2*, *LAG3*, *TIGIT*, *PDCD1*, *HAVCR2*, and *CTLA4* were found to be mostly enriched in CAYA patients (Figure 3B).

Next, we conducted a pseudotime trajectory analysis on CD8+ T cells using the Monocle 3^[^ ^15^ ^]^ (Figure 3C). Along the pseudotime trajectory, we observed that cytotoxicity‐related genes (e.g., *KLRG1*, *GNLY*, and *GZMH*) were gradually downregulated while dysfunction‐related genes (e.g., *CTLA4*, *LAG3*) were gradually upregulated^[^ ^14^, ^23^, ^24^ ^]^ (Figure 3D). This pseudotime trajectory recapitulated the progression of CD8+ T cells from a cytotoxic state through a predysfunctional state to a fully dysfunctional state, along with an escalating degree of exhaustion. We further investigated the transcriptional changes associated with transitional states and categorized the CD8+ T cell clusters into three phases (Figure 3E). CD8T_Tn cells (including Tn_CCR7, Tn_CD55, and Tn_PLCG2) were predominantly in state 3, characterized by upregulated expression of *CCR7*, *ANXA1*, *TCF7*, *LEF1*, and *KLF2*, indicating the lowest cytotoxic capacities.^[^ ^18^, ^25^ ^]^ In another trajectory branch (state 2), CD8+ T cells progressed from Tnaive‐Tc17 to Tem and then to Tex. STARTRAC pairwise transition analysis based on TCR sharing further confirmed that Tem_GZMK cells have a strong propensity to transition into Tex cells, supporting the hypothesis that CD8+GZMK+T cells, considered a “pre‐exhausted” state within tumors, can progress into terminally exhausted T cells (Figure 3G).^[^ ^26^, ^27^ ^]^ Cells in the end of trajectory demonstrated the highest expression levels of *PDCD1*, *LAG3*, *NFATC1*, and *CXCR5*, positively correlated with *TOX2*.^[^ ^28^, ^29^ ^]^ Conversely, *IL2*, *IFNG*, and *HAVCR2*, negative correlated with *TOX2*, were downregulated during pseudotime. Notably, the expression of these Tex‐related genes was higher in CAYA patients than in adults, indicating a stronger suppressive role of Tex in CAYA patients (Figure 3F). Similar to CD4+ T cells, cytotoxicity score (0.003 ± 0.352 vs −0.08 ± 0.345) was higher in adult patients, while exhaustion (0.031 ± 0.173 vs 0.046 ± 0.179), apoptosis (0.084 ± 0.03 vs 0.136 ± 0.03), and migration (0.061 ± 0.036 vs 0.1 ± 0.032) score were higher in CAYA group (Figure 3H). Among them, important indicators related to exhaustion, such as *PDCD1*, *CTLA4*, and *LAG3* were mainly highly expressed in Tex and pediatric cases (Figure 3I; Figure S3B, Supporting Information). The results were verified by mxIHC of PDCD1/CD3 and CTLA4/CD3 (Figure S3C, Supporting Information). More importantly, through Scenic analysis, we found that *SMAD1*, *BACH1*, and *IRF4* were enriched in Tex cells, and may play an important role specifically in terminally exhausted T cells (Figure 3J).

In summary, pseudotime trajectory analysis revealed a progression from cytotoxic to exhausted states, with CAYA patients exhibiting higher levels of proexhaustion factors such as *TOX2* and *PDCD1*, suggesting a stronger suppressive tumor microenvironment.

### 2.4 Immune and Endothelial Cell Infiltration in PTC

Since we observed remarkable divergence between CAYA and adult patient ecosystems, we set out to identify disease‐specific cell types in further depth. Subclustering of NK cells led to the identification of 3 clusters including NK_CD56, NK_CD16, and NKT, with NK_CD56 cells dominating in CAYA tumor tissues but showing lower cytotoxicity and activation score, potentially contributing to the higher metastatic tendency in children (Figures S4A–E and S6 and Table S3, Supporting Information). Subclustering of B cells identified 4 distinct clusters: B_naive, B_memory, B_GC, and B_plasma. B_naive cells were more abundant in adult tumor tissues and adjacent normal tissues compared to those from CAYA patients. In contrast, B_plasma and B_GC cells were more enriched in CAYA tumor and adjacent tissues than in adults (Figures S4F–H and S6 and Table S3, Supporting Information). B cell receptor score (BRs) in the germinal center demonstrated the highest level, followed by B memory cells. The BRs of CAYA patients were significantly higher than those of adults (Figure S4I, Supporting Information). Kyoto Encyclopedia of Genes and Genomes (KEGG) pathway analysis also indicated that the B cell receptor signaling pathway in CAYA was significantly more active than that in adults (Figure S4J, Supporting Information). Subclustering of myeloid identified 12 clusters clearly delineating macrophage, monocyte, dendritic cells, and neutrophil cells. Tumor‐associated macrophage (TAM) exhibited higher infiltration in tumor tissues than in normal tissues (Figure S5A–D, Supporting Information; Figure 6C). Flow cytometry showed that the proportion of CD206+M2 cells in CAYA‐PTC tumor tissues (64.9 ± 17.9%) was significantly higher than in adults (35.4 ± 11.2%, *P* < 0.05), and significantly higher than in adjacent tissues (9.9 ± 7.4%, *P* < 0.05) (Figure S6 and Table S3, Supporting Information). TAM5_FOLR2 had high M2 score and low phagocytic score, indicating TAM5_FOLR2 is located at the end of macrophage differentiation (Figure S5E,F, Supporting Information). The M2 score of CAYA patients is higher than that of adults, but the phagocytic score is lower (Figure S5E,F, Supporting Information). This suggests that myeloid cells in children have insufficient phagocytic ability and a tendency to favor M2 cells related to tumor suppression, which is an important factor in the tendency of CAYA patients to metastasize. Subclustering of endothelial cells (ECs) identified six clusters, mainly categorized into vascular and lymphatic endothelium, showed increased infiltration of capillary EC cells in CAYA tumors, likely contributing to angiogenesis (Figure S5G–J, Supporting Information). Overall, immune cell infiltration profiles, particularly involving NK and myeloid cells, highlighting the unique tumor microenvironment and may influence tumor progression and metastasis in pediatric patients.

### 2.5 Intratumor Heterogeneity and Differences in Cell Differentiation of Thyrocytes between CAYA and Adult Patients with PTC

**Figure 4:**
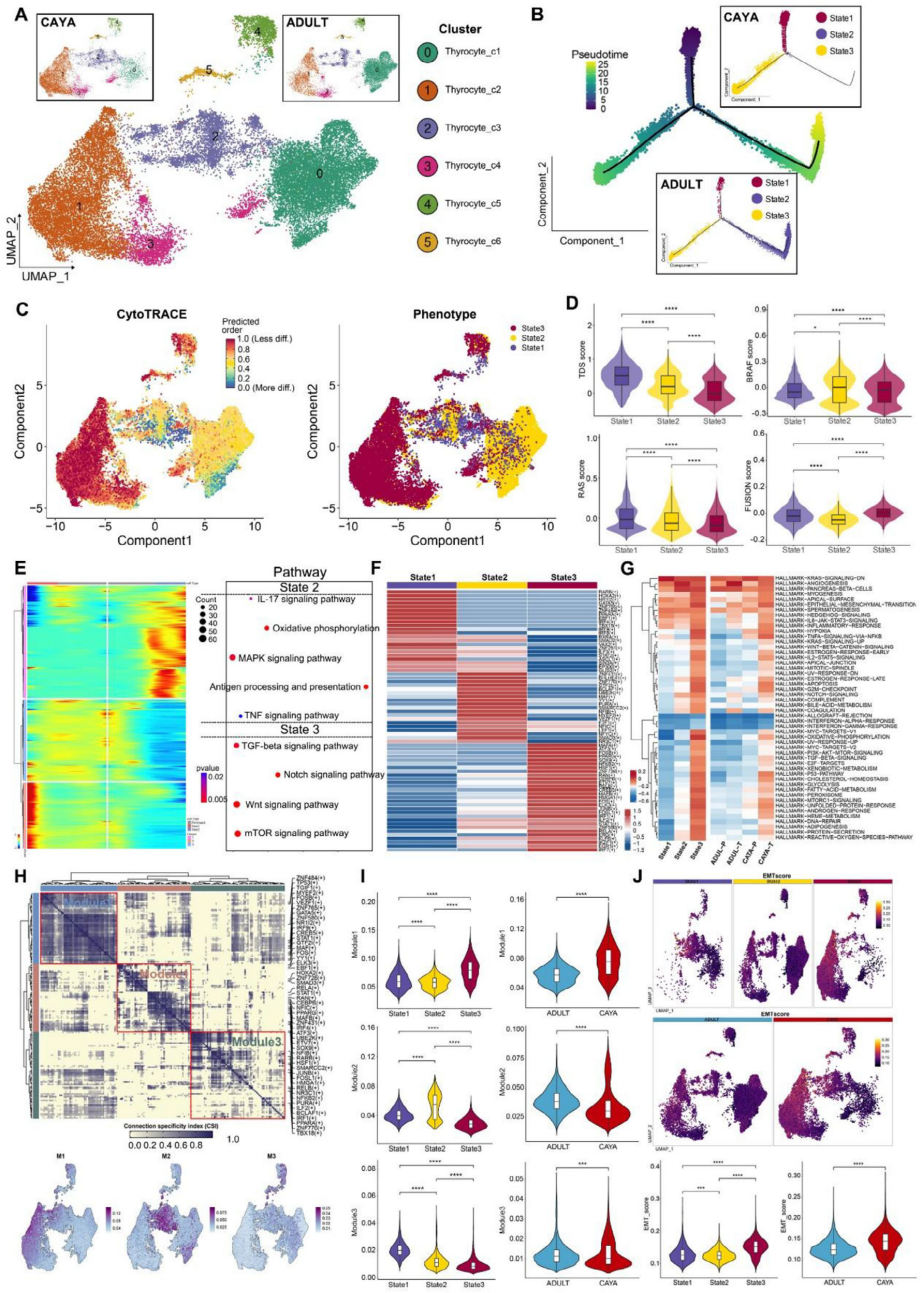
EMT contributes vital important role in CAYA patients. A) UMAP of thyrocytes, with split to CAYA patient (top left) and adult patient (top right). B) Developmental trajectory of thyrocytes, colored by states (middle) with split to CAYA patient (top right) and adult patient (middle bottom). C) Predicted UMAP mapping by CytoTRACE, which orders thyrocytes based on their developmental potential from more differentiation (lowest values) to less differentiation (highest values) (upper) and UMAP of thyrocytes colored by states (lower). D) Violin and box plots showing significant differences in TDS scores, BRAF scores, RAS scores, and Fusion scores of the three states. The middle lines of the box plots show the median (central line), the lower and upper hinges show the 25–75% interquartile range (IQR). *P < 0.05, **P < 0.01, ***P < 0.001, ****P < 0.0001; unpaired two‐sided *t*‐test. E) Beam analysis showing the gene expression profiles of thyrocytes at branchpoint. F) Heatmap of TF (transcription factor) activities in the three thyrocyte states, scored by SCENIC. G) Differences in the activities of hallmark pathways between different thyrocyte states, scored by GSVA. H) Identified regulon modules based on regulon connection specificity index (CSI) matrix, along with representative transcription factors and corresponding binding motifs. UMAP plots showing the distribution of three modules reported by regulon modules analysis (bottom). I) Violin and box plots of CSI scores for each module in three states and two groups of patients. *P < 0.05, **P < 0.01, ***P < 0.001, ****P < 0.0001; unpaired two‐sided *t*‐test. J) UMAP mapping and violin–box plots of EMT score in three states and two groups of patients.

To systematically interrogate the intrathyrocytic heterogeneity of PTC, we acquired a total of 26 933 cells from these samples following standard procedures, finally six thyrocyte subclusters were annotated (**Figure** **4**A).

We next explored the dynamic states and cell transitions in thyrocytes by inferring state trajectories using Monocle 2.^[^ ^30^ ^]^ This analysis showed three states across all patients. Notably, thyrocytes in the CAYA group are predominantly in state 1 and 3, whereas all three states were present in adults^[^ ^6^ ^]^ (Figure 4B). This indicates different differentiation trends between CAYA and adult patients. To further characterize these states, we conducted a CytoTRACE analysis, predicting that state 3 cells have less developmental potential than state 1 and 2 cells (Figure 4C). The cells with the highest CytoTRACE score, indicating the most immature cells, mapped to the endpoint of the trajectory by Monocle 2. Additionally, the TDS scores were consistent with CytoTRACE predictions (Figure 4D). State 2 cells exhibited the highest BRAF score, moderate TDS, RAS, and FUSION scores, and were predominant in adults, suggesting these cells may represent milder tumor phenotypes (mild‐state, BRAF‐like). Conversely, state 3 cells had the lowest TDS score and the highest FUSION score, indicating a poor differentiation degree likely related to gene fusion events (Fusion‐like). This suggests that in adult thyroid cancer, normal state 1 cells can progress to moderately differentiated state 2 cells or less differentiated state 3 cells. In CAYA PTC, however, normal thyroid follicular cells tend to progress directly to poorly differentiated state 3 cells. To understand the mechanisms underlying these different differentiation tendencies, we performed branch analysis at the trajectory branchpoint. We identified a cluster of genes highly expressed in state 2, including several MAPK signaling‐related genes, IL‐17 signaling pathway and TNF signaling pathway. Additionally, TGF‐beta, Notch, Wnt, and mTOR signaling pathway were upregulated in state 3 (Figure 4E).

State 3 thyrocytes preferentially upregulated dedifferentiation‐related TFs and pathways (Figure 4F,G), while simultaneously exhibiting the lowest levels of thyroid epithelial markers (*TG*, *TPO*, *TFF3*, *DIO2*, *ID4*). Transcription factors often work in combination to coordinate gene expression. To systematically characterize these combinatorial patterns, we compared the atlas‐wide similarity of regulon specificity score (RSS) scores for each regulon pair based on the Connection Specificity Index (CSI). Strikingly, these 202 regulons organized into three major modules (Figure 4H). Module M1 contains regulators *ELK3*,^[^ ^31^ ^]^ *EBF1*, *STAT1*,^[^ ^32^ ^]^ and *CEBPB*,^[^ ^33^ ^]^ which are essential for metastasis. M3 includes a mix of grow factors, such as *ATF3*, *ETV7*, and *FOSL1*.^[^ ^34^ ^]^ For each module, we identified several representative regulators through their average activity scores. Notably, the regions mapped by each module on UMAP corresponded to special states, for instance: Module 1, Module 2, and Module 3 aligned with state 3, state 2, and state 1, respectively (Figure 4H). The regulon activity scores across various states also confirm this alignment (Figure 4I). To further explore the role of epithelial–mesenchymal transition (EMT) in thyrocytes, we found that the EMT score was highest in state 3, and the EMT score in the CAYA group was higher than that in adults (Figure 4J).

Notable differences in gene expression and differentiation trends were observed between CAYA and adult groups. Trajectory analysis revealed state 3, marked by poor differentiation and high FUSION scores, emerged as a key endpoint for CAYA thyrocytes, potentially driven by gene fusion events. Importantly, EMT was implicated as a critical factor in the progression of state 3 cells, particularly in CAYA. These findings highlight distinct cellular dynamics and signaling pathways in CAYA and adult PTC.

### 2.6 Identification and Molecular Characterization of Four CAF Subtypes in PTC

**Figure 5:**
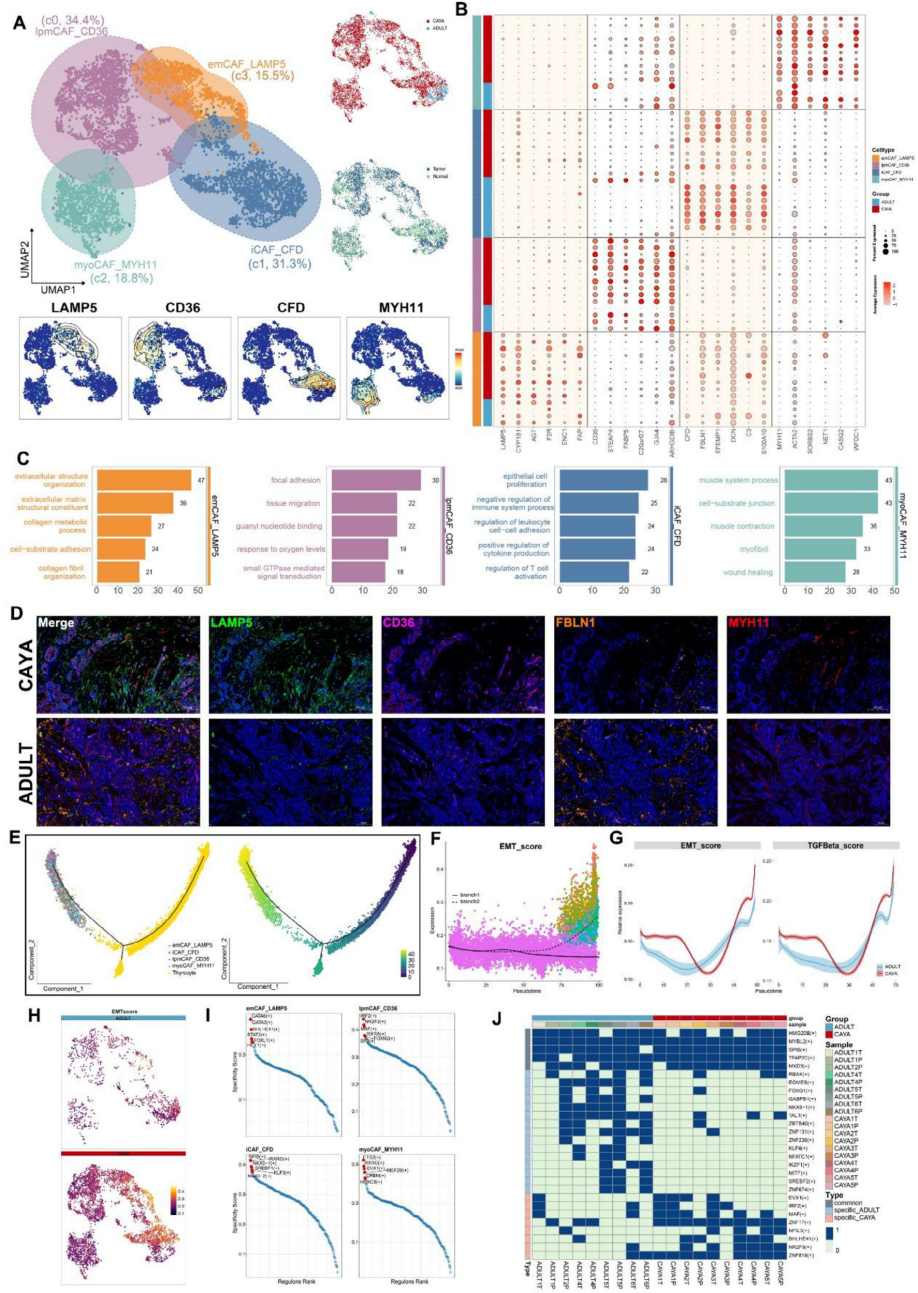
Subtyping of cancer‐associated fibroblasts and their heterogeneity between CAYA and ADULT. A) UMAP plots for the four different CAF subtypes in papillary thyroid tumors (top left), and each cell colored for group (top right), tissue origin (bottom left). UMAP representations with cells colored by the expression level of marker genes of PTC. The black lines represent the spatial density of the cells expressing the given gene higher than the mean level of expression (bottom). B) Bubble plot showing top six differentially expressed genes in each CAF subtypes and patient. The size of the dot indicates the fraction of cells expressing a particular marker, and the intensity of the color represents the level of mean expression. Cellular phenotypes and age group are indicated left side the dot map. C) Bar plot showing the gene count for the most significantly upregulated GO BP pathways in each subtype, calculated through GSVA and Empirical Bayes Statistics for differential expression. D) Multiplex immunofluorescence staining showed major CAF clusters existed in PTC tissues. A pseudocolored image depicting different markers identified by mxIHC (colored as indicated in the key) and the results from histocytometry‐based cell classification (anti‐LAMP5 for emCAF_LAMP5, anti‐CD36 for lpmCAF_CD36, anti‐SMMC for myoCAF_MYH11, anti‐FBLN1 for iCAF_CFD). E) The pseudotime trajectory analysis of thyrocytes and fibroblasts inferred by Monocle 2. Each color represents one cell subtype (left), each point corresponds to one single cell (right). F) Trace plots of the EMT score along pseudotime separated by branch point. G) Shaded line plot indicating the expression levels of the EMT score (left) and TGF‐β (right) along the pseudotime in CAYA and adult patients. H) UMAP reflection of EMT scored by Ucell. I) Rank plot of TF (transcription factor) activities in the four CAF types, scored by pySCENIC. J) The binomial distribution heatmap of transcription factors of age‐specific transcription factors in CAYA and adults.

To identify and classify CAF types, we investigate fibroblast phenotypic heterogeneity in‐depth. We performed unsupervised hierarchical clustering of the single‐cell gene expression profiles of fibroblasts, identifying four clusters (**Figure** **5**A). All four subclusters expressed high levels of canonical fibroblast markers, such as *TAGLN* and *RGS5*, confirming their identity as fibroblasts. However, each subcluster displayed distinct transcriptomic signatures (Figure 5B; Figure S7A, Supporting Information).

**Figure 6:**
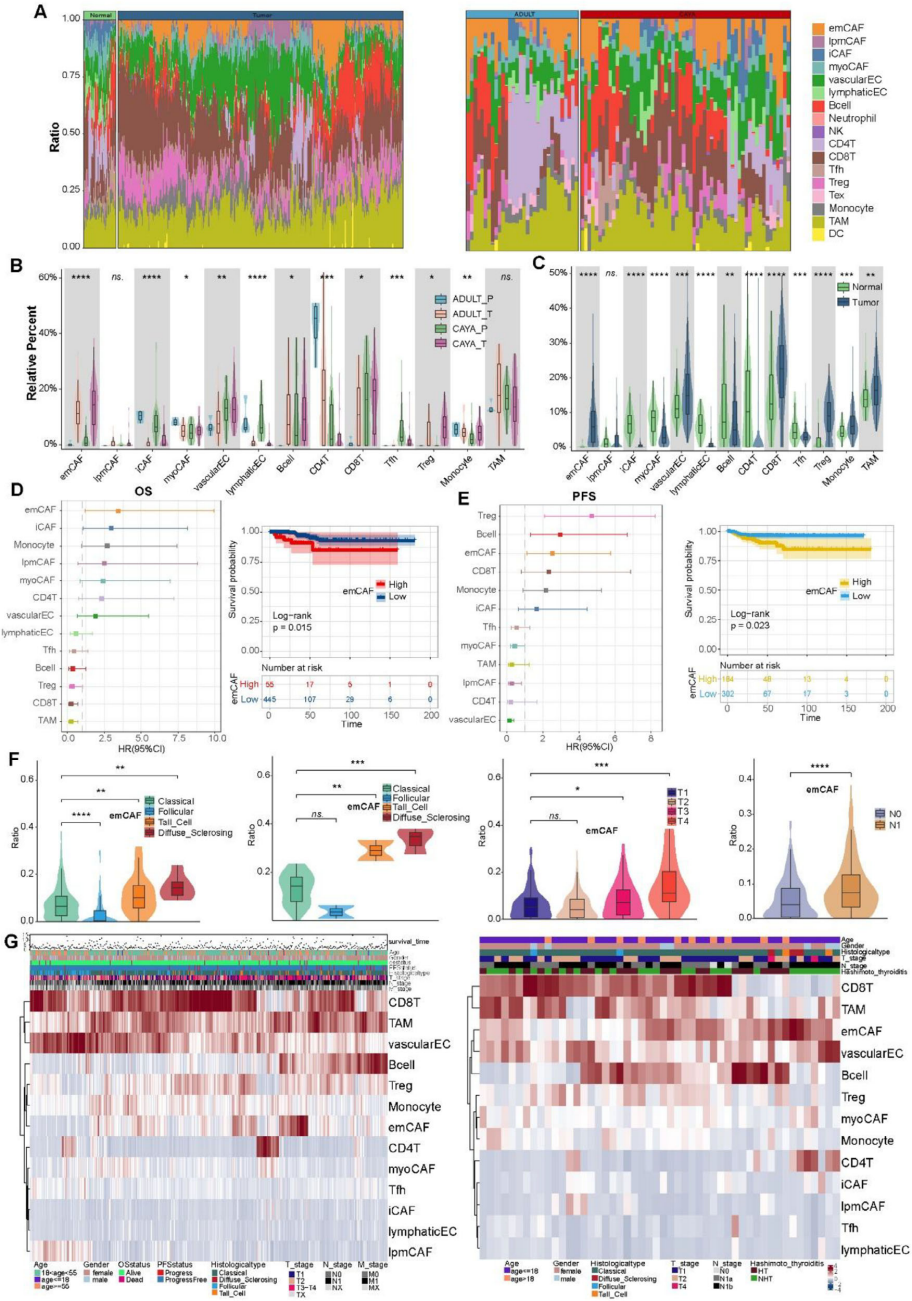
Cell subtypes of bulk RNA‐seq and the relationship with clinical features. A) Bar plot of cell abundance predicted per sample from TCGA‐THCA and bulk RNA sequencing cohort by CIBERSORTx. B) Box plot showing the relative abundance of cell types for CAYA and adult in tumor and normal tissue in bulk RNA‐seq cohort. C) Box plot showing the relative abundance of cell types in tumor and normal tissues in TCGA‐THCA cohort. *P < 0.05, **P < 0.01, ***P < 0.001, ****P < 0.0001; two‐sided *t*‐test. D) Association between relative cell abundance and patient overall survival (OS) from TCGA‐THCA cohort (left) and Kaplan–Meier overall survival curves for emCAF (right). E) Association between relative cell abundance and patient progress free survival from TCGA‐THCA cohort (left) and Kaplan–Meier progress free survival curves for emCAF (right). HR, hazard ratio. Multivariate Cox regression. *P*‐value was determined by Kaplan–Meier survival curves and log‐rank test. F) Violin plot of the relationship between the proportion of emCAF cells and pathological subtypes in TCGA‐THCA database and in bulk RNA sequencing data, T stage, and N stage. Error bar: mean value ± sd. *P < 0.05, **P < 0.01, ***P < 0.001, ****P < 0.0001; *P* values were determined by two‐side Student's *t* test. G) Heatmap of cell abundance predicted per sample from TCGA‐THCA cohort (left) and bulk RNA sequencing data (right) by CIBERSORTx. Shown are row *z*‐score.

Subcluster 3 CAFs accounted for 15.5% and expressed high levels of *LAMP5* and *FAP* (Figure 5A,B). GO terms enrichment and Gene Set Variation Analysis (GSVA) leading us to term this subcluster extracellular matrix CAFs (emCAF_LAMP5) (Figure 5C; Figure S7B, Supporting Information).^[^ ^35^ ^]^ emCAF_LAMP5 was significantly increased in CAYA patients, with activated angiogenesis and P53 pathways, may be associated with the more aggressive clinical manifestations observed in CAYA PTC patients (Figure S7D–F, Supporting Information; **Figure** **6**A–C). A high accumulation of emCAFs was positive related to poorer overall survival (OS) and poor progress free survival (PFS) (Figure 6D). Moreover, higher emCAF infiltration indicates a greater tendency to develop into diffuse sclerosing papillary thyroid cancer, larger tumor diameter and lymph node metastasis (Figure 6F,G). More interestingly, emCAF_LAMP5 was particularly abundant in tumor samples from CAYA2 and CAYA4, both of whom had diffuse sclerosing papillary thyroid carcinoma (DS‐PTC) based on paraffin pathology. This suggests that emCAF_LAMP5 may be associated with DS‐PTC subtype, which is more prevalent in CAYA patients.^[^ ^36^ ^]^ Clinically, DS‐PTC patients exhibit faster tumor progression and higher invasiveness, and a high proportion of emCAF_LAMP5 may be the underlying mechanism in CAYA patients^[^ ^37^ ^]^ (Figure S7E, Supporting Information). Subcluster 0 CAFs, which constituted the majority of the CAF populations (34.4%), expressed high levels of lipid metabolism‐related genes (*CD36*, *STEAP4*, and *FABP5*, Figure 5B), aligns with previously reported findings and was therefore named lipid matrix‐related CAF (lpmCAF_CD36).^[^ ^38^ ^]^ iCAF_CFD (c1, 31.3%) comprising the second largest group (31.3%), were characterized by high expression levels of inflammatory related genes and complement pathway, including *CFD*, *FBLN1*, and *C3*.^[^ ^39^ ^]^ This cluster was higher in CAYA normal tissues, closely related to immune response activation regulation, suggesting that the immune regulatory function of the TME in children may be weaker (Figure S7D–G, Supporting Information; Figure 6A–C). Subcluster 2 (18.8%) exhibited high differential expression levels of *MYH11*, *MYH9*, and *ACTA2*. GSVA indicated strong upregulation of myogenesis, consistent with our annotation of these cells as myoCAFs (myoCAF_MYH11) (Figure 5B,C; Figure S7B, Supporting Information).^[^ ^40^ ^]^

**Figure 7:**
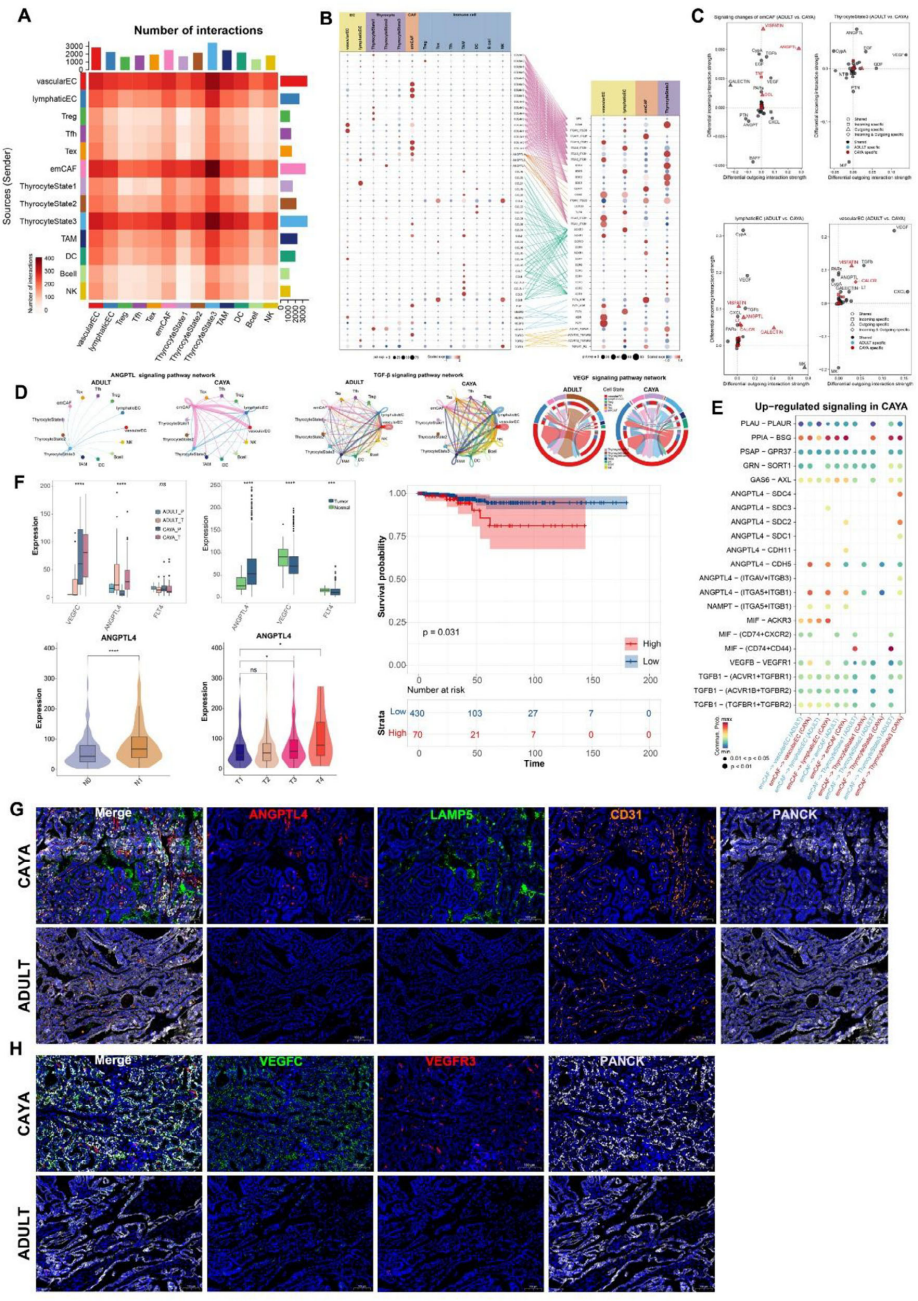
Complex cell–cell communication networks in the PTC TME. A) Heatmap showing the strength of incoming and outgoing events in interactions between different clusters in the PTC. Histograms separately count the overall intensity of outgoing (*y*‐axis) and incoming (*x*‐axis) events for each cluster. B) Dot plots show gene expression levels of receptor–ligand pairs involved in interactions between different clusters in PTC. C) Interaction strength of emCAF, thyrocyte State 3, lymphatic EC, and vascular EC subclusters incoming and outgoing events between CAYA and adult PTC. D) Circle and chord diagram showing predicted cell–cell interactions of ANGPTL, TGF‐β, and VEGF signaling pathway between CAYA and adult PTC. The arrow width indicates the interaction strength levels. E) Bubble heatmap showing the mean interaction strength for selected ligand–receptor pairs between CAYA and adult in various cell–cell clusters. Dot size indicates *P*‐value generated by permutation test, colored by interaction strength levels. F) Box plots showing the expression level of VEGFC, ANGPTL4, and FLT4 from PTCs in TCGA cohort between CAYA and adult. Violin plot showing expression of ANGPTL4 expression among different N and T stages. *P < 0.05, **P < 0.01, ***P < 0.001, ****P < 0.0001; two‐sided *t*‐test. Kaplan–Meier plots for overall survival of ANGPTL4‐high and ‐low in PTC patients. *P*‐value was determined by Kaplan–Meier survival curves and log‐rank test. G) Representative micrographs from multiplexed IHC (mxIHC) labeled by ANGPTL4, LAMP5, CD31, and PANCK performed on serial sections. H) Representative images from multiplexed IHC (mxIHC) labeled by VEGGC and VEGFR3 performed on serial sections.

Guided by our scRNA‐seq data, we next used mxIHC by a five‐labeled antibody panel to analyze the spatial distributions of our defined CAF phenotypes in PTC samples (the detail see “Multiplex Immunohistochemistry” in the Experimental Section) (Figure 5D). The panel also included *COL1A1* to delineate the boundary of the extracellular matrix (not shown). Consistent with our scRNA‐seq findings, emCAF_LAMP5 was predominant in CAYA patients and significantly higher than in adults. Conversely, iCAF_CFD subtypes were mainly present in adult patients, as well as in adjacent normal tissues (Figures 5D and 7F,G; Table S4, Supporting Information).

EMT enhances the malignant characteristics of cancer cells, including invasive behavior, tumor stem cell activity, and greater resistance to chemotherapy and immunotherapy.^[^ ^41^, ^42^ ^]^ To investigate EMT in PTC and potential differences between CAYA and adult patients, we extracted thyrocytes and CAFs from tumor tissue and performed trajectory analysis (Monocle 2). The results showed that thyrocytes could transform into CAFs over pseudotime (Figure 5E). During this process, the EMT score in branch 2 (Figure 5F) and the expression of thyrocyte marker genes such as *CD24*, *TGF‐β*, and *TPO* gradually decreased, while the expression of CAF marker genes such as *TAGLN*, *COL1A1*, and *RGS5* increased (Figure S7H, Supporting Information). Notably, the EMT score and *TGF‐β* levels in CAYA were higher than in adults (Figure 5G,H; Figure S7I, Supporting Information). Among the four CAF subtypes, emCAF_LAMP5 had significantly higher EMT scores than the other three CAFs (Figure S7I, Supporting Information). Through the coculture experiment of thyroid cancer cells and TGF cytokines, we found that under the stimulation of TGF, the morphology of thyroid tumor cells changed, and N‐cadherin and vimentin increased, while β‐catenin and Claudin‐1 decreased (Figure S7J,K, Supporting Information). These findings suggest that PTC in CAYA is more susceptible to microenvironmental remodeling through the EMT process, promoting metastasis and stemness.

To comprehensively reconstruct the gene regulatory networks for four CAFs, we applied the SCENIC pipeline to analyze the data. For each regulon, we evaluated its activities associated with each of the subclusters and defined an RSS based on Jensen–Shannon divergence.^[^ ^43^ ^]^ We then selected the regulons with the highest RSS values and further examined their functional properties. Our network analysis identified *GATA6*, *GATA3*, *BHLHE41*, *STAT2*, *FOXL1*, and *HEY1* as the most specific regulons associated with emCAF_LAMP5 (Figure 5I). Notably, the GATA family regulates EMT, promotes fiber formation, and played important role in tumorigenesis and development.^[^ ^44^ ^]^ *STAT2* regulates glycolysis and promotes angiogenesis, and its specific elevation promotes the protumorigenic function of emCAF_LAMP5.^[^ ^45^ ^]^ Using the binomial value results of SCENIC, we determined whether a particular regulon is specific or shared by the number of times it occurs in the sample, thus identifying the specific or shared regulons of CAF in CAYA and adults (Figure 5J). It can be seen that the most specific regulons including *MAF*, *ZNF17*, *BHLHE41*, *NR2F6*, and *ZNF816*, which may contribute to the different transcriptional profiles between CAYA and adult patients (Figure 5J).

Overall, emCAF_LAMP5, enriched in CAYA patients, exhibited high EMT and angiogenesis activities, potentially contributing to the aggressive clinical behavior observed in CAYA PTC. Trajectory analysis revealed thyrocytes transitioning into CAFs, with EMT scores and *TGF‐β* levels higher in CAYA. SCENIC network analysis identified key regulons driving the distinct transcriptional profiles and protumorigenic functions of CAF subtypes, with emCAF_LAMP5 regulated by *GATA6*, *STAT2*, and others. These findings underscore the microenvironmental differences in PTC between CAYA and adults, particularly in EMT and CAF‐mediated tumor progression.

### 2.7 Crosstalk among Tumor and Tumor‐Infiltrating Cells in PTC

To further gain insights into the regulatory relationships among cell subclusters, ligand–receptor (L–R) pair interactions were investigated using CellPhoneDB. We identified different cell–cell interaction models in CAYA and adult patients (Figure S8A–C, Supporting Information). In CAYA samples, we observed enhanced interactions between lymphatic ECs and multiple cell types, including Thyrocyte_State3, CAFs, and ECs. This pattern is consistent with the critical role lymphatic ECs play in driving tumor progression within the CAYA tumor microenvironment. In contrast, interaction of lymphatic ECs with other cells was only slightly enriched in adult patients (Figure S8C, Supporting Information).

Next, we performed CellChat analysis to identify cell–cell interactions in the PTC TME. Focusing on the interactions involving CAFs, we first investigated the connections with thyrocytes, ECs, and immune cells. It appears that emCAF, vascular EC, lymphatic EC, and thyrocyte State 3 mediated the most efferent and afferent events from other subpopulations, possibly representing a signaling center and secretory hub within the tumor (**Figure** **7**A). Most importantly, emCAF interacted with thyrocyte State 3 in EMT related signaling pathway in COLLAGEN, FN1, and PERIOSTIN (Figure S8D–F, Supporting Information). emCAFs expressed the highest ligands in COLLAGEN pathway, such as *COL6A1‐3* and *COL1A1‐2*. They also highly expressed ligands involved in downstream pathways, including *GP6*, *ITGA3_ITGB1*, *SDC2*, and *SDC1* (Figure 7B). emCAF subclusters seem to provide costimulatory signals to thyrocyte State 3 cells through *ANGPTL4‐CDH11*/(*ITGAV_ITGB3*) and *TGFB3*‐(*ACVR1_TGFbR*)/(*TGFbR1_R2*) interactions. Potential *VEGFC*‐(*FLT4/KDR*) interaction was detected between vascular EC and thyrocyte State 3. Additionally, stronger coinhibitory signals from lymphatic EC to thyrocyte State 3 and emCAF cells were likely mediated by the *CCL21*‐*ACKR4* and *CCL21*‐*CCR7* axes (Figure 7B).

In addition, we calculated the incoming and outgoing interaction strengths of cells and found that *ANGPTL*, *VEGF*, and *TGF‐β* exhibited high signal in CAYA patients in emCAF, vascular EC, lymphatic EC, and thyrocyte State 3^[^ ^46^ ^]^ (Figure 7C). We hypothesized that CAYA display distinct subcellular interaction relationships within the TME, which may profoundly influence the tumor phenotype. To test this hypothesis, we performed cell–cell communication analysis separately in CAYA and adult patients, focusing on *ANGPTL*, *TGF‐β*, and *VEGF* signaling pathway. Notably, emCAFs had more interactions with lymphatic EC, vascular EC, and thyrocyte State 3 in CAYA (Figure 7D). Moreover, *ANGPTL4* and related ligand genes seems more common in CAYA (Figure 7E). We subsequently focused on *ANGPTL4*, *FLT4* (*VEGFR3*), and *VEGFC* expression in TCGA database and our whole transcriptome sequencing data by comparing clinical information. *VEGFC* was significantly higher in CAYA patients, and *ANGPTL4* was highly expressed in tumors, with greater expression in CAYA tumor tissues. *ANGPTL4* also exhibited high levels in N1 and more progressed tumor patients (Figure 7F).

To orthogonally validate the emCAF, vascular EC, lymphatic EC, and thyrocyte subpopulations identified by scRNA‐seq, we designed an mxIHC panel with four markers (for the detail see “Multiplex Immunohistochemistry (MxIHC)” in the Experimental Section). Consistent with previously defined nomenclature for the phenotypes, our mxIHC analysis showed the subpopulations were accurately identified within the respective regions. We examined the relative abundance of each subpopulation in CAYA and adult tissue samples. mxIHC confirmed that *ANGPTL4* and *LAMP5* were prevalent in CAYA. Furthermore, emCAFs in CAYA samples were found in closer proximity to *CD31*, indicating that *ANGPTL4* expression in emCAFs promotes angiogenesis in CAYA PTC (Figure 7G; Figure S7G, Supporting Information). Additionally, the expression of *VEGFC* on the surface of CAYA tumor cells was significantly higher than in adult samples and was correlated with VEGFR3+ lymphatic ECs, confirming the prolymphangiogenic effect of tumors in CAYA patients (Figure 7H; Figure S7G, Supporting Information).

emCAFs in CAYA prominently expressed *ANGPTL4*, and *TGF‐β* ligands, promoting angiogenesis and EMT, as validated by transcriptomic and mxIHC analyses. *ANGPTL4* and *VEGFC* were significantly upregulated in CAYA, suggesting a proangiogenic and prolymphangiogenic TME driving more aggressive tumor phenotypes (thyrocyte State 3) in these patients. These findings highlight the critical role of specific cellular interactions and signaling pathways in shaping the distinct TME of CAYA PTC.

### 2.8 FAP Is a Promising Diagnostic Target in Children

**Figure 8:**
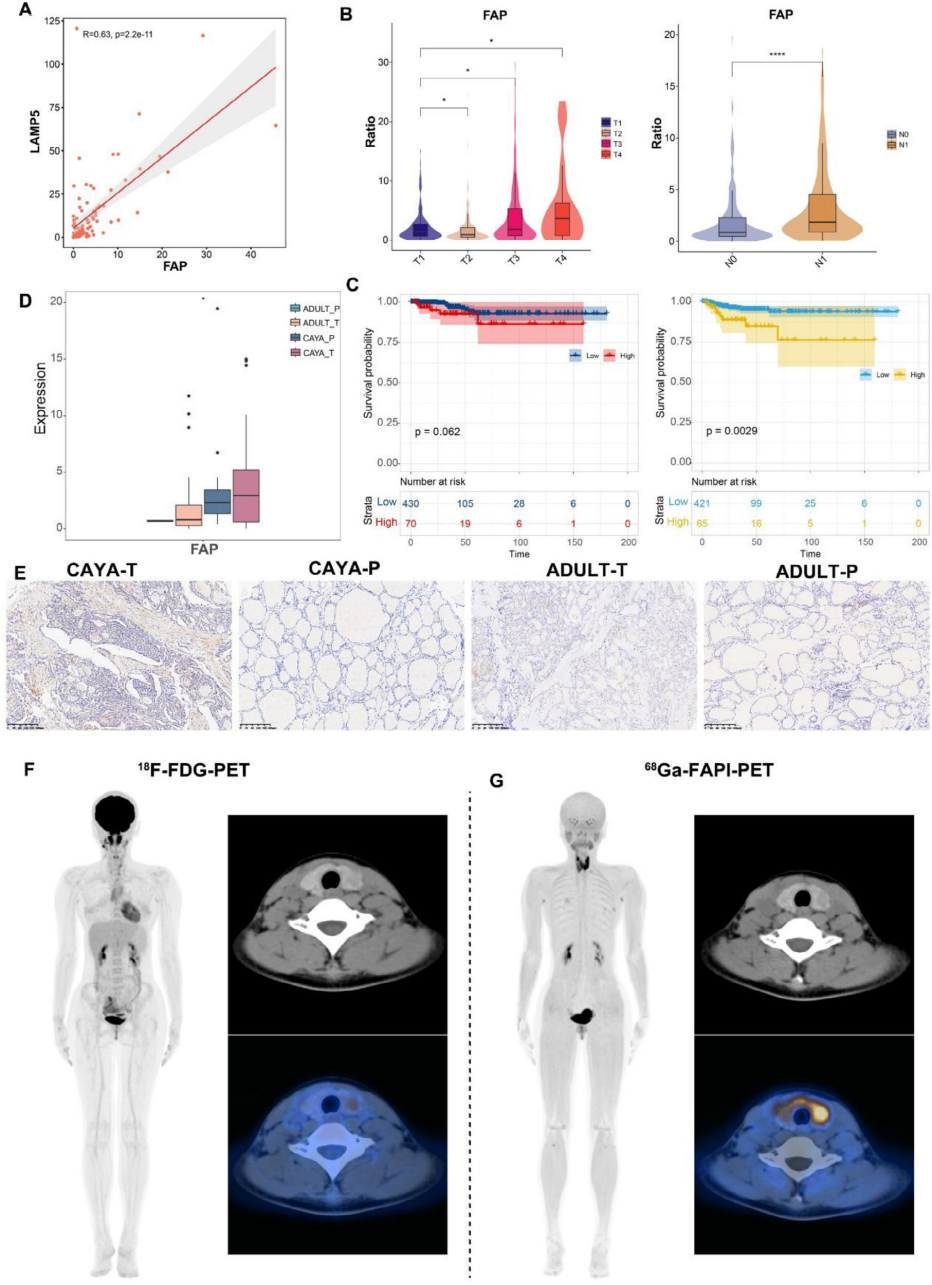
Characteristics of FAP and serves as a target for PET imaging. A) Dot plot showing the correlation between FAP and LAMP5. The *P* value was generated by Pearson correlation. B) Violin plot of the relationship between the proportion of FAP and T stage (left) and N stage (right) in TCGA‐THCA database. *P < 0.05, **P < 0.01, ***P < 0.001, ****P < 0.0001; two‐sided *t*‐test. C) Overall survival curves (left) and Kaplan–Meier progress free survival curves (right) for FAP of the patients with PTC in TCGA‐THCA cohort for stratified by subclusters’ abundance using the optimal cut‐point for dichotomization. Statistical significance P value was assessed using log‐rank test. D) Related expression of FAP in CAYA and adult patient separated by tumor and para‐tumor tissues staining registration for FAP in the tumor and para‐tumor tissue from CAYA and adult patient (n=3). Overall survival curves (left) and Kaplan–Meier progress free survival curves (right) for FAP of the patients with PTC in TCGA‐THCA cohort for stratified by subclusters’ abundance using the optimal cut‐point for dichotomization. Statistical significance *P*‐value was assessed using log‐rank test. F) Comparation of ^18^F‐FDG‐PET and ^68^Ga‐FAPI‐PET imaging.

In the scRNA‐seq analysis of CAF subpopulations, we found that the emCAF_LAMP5 subpopulation was highly expressed in CAYA PTC, with *FAP* protein also highly expressed in this population (Figures 5B and 8A). Immunohistochemistry (IHC) staining confirmed that *FAP* expression was increased in both CAYA and adult tumor tissues and was mainly expressed in the mesenchyme (**Figure** **8**B). Besides, in bulk‐RNA sequencing data, *LAMP5* and *FAP* expression were positively correlated (Figure 8C). Consistent with *LAMP5*, the high expression of *FAP* was closely related to tumor size, lymph node metastasis, and poorer prognosis (Figure 8D,E). The *FAP* inhibitor (FAPI), which targets *FAP*, can be used as a radiotracer and has been used in PET imaging. By binding to *FAP*, FAPI can visualize the location and size of tumors on PET‐CT scans, aiding in determining tumor type and location and guiding the selection of tumor therapy. FAPI‐PET is considered a novel tumor imaging agent with potential applications.^[^ ^47^, ^48^ ^]^ FAPI‐PET has been demonstrated in thyroid tumors for diagnostic use and may have advantages over conventional FDG‐PET.^[^ ^49^, ^50^ ^]^ And FAPI has also been shown to be therapeutic in metastatic radioiodine‐refractory thyroid cancer.^[^ ^51^ ^]^ Given the high proportion of FAP+CAF in pediatric tumors, we believe that FAPI‐PET may be more diagnostic for pediatric thyroid cancer. In a 12‐year‐old patient with preoperatively diagnosed CAYA PTC, ^68^Ga‐FAPI‐PET showed higher visualization compared with ^18^F‐FDG‐PET (Figure 8F,G). This preliminary observation from a single pediatric case suggests that FAPI may represent a superior diagnostic and targeted therapeutic option for pediatric PTC patients in the future.

In summary, this study constructed a comprehensive single‐cell atlas of papillary thyroid carcinoma, systematically analyzing the tumor microenvironmental heterogeneity between CAYA and adult patients. The findings revealed higher proportions of immunosuppressive T cells (Treg/Tfh) and exhausted T cells (CD8+ Tex) in CAYA patients. In addition, these patients showed enhanced epithelial–mesenchymal transition and increased angiogenesis. There was also significant enrichment of the CAF subtype emCAF_LAMP5, which is closely associated with more aggressive clinical phenotypes. Importantly, we discovered three phenotypes of thyrocytes populations, which shape bulk molecular subtypes and tumor characteristics. CAYA‐PTC patients lack a “mild‐state (BRAF‐like, state 2)” malignant thyrocyte population, indicating that normal thyroid follicular cells tend to progress directly to poorly differentiated state 3 cells. Through detailed pathway analysis and cell–cell communication modeling, significant activation of ANGPTL, VEGF, and EMT‐related pathways was identified in CAYA patients, leading to increased lymphatic vessel proliferation and angiogenesis in CAYA tumor, thereby promoting tumor metastasis. Moreover, FAP was highlighted as a promising diagnostic and therapeutic target, underscoring ^68^Ga‐FAPI‐PET as a superior diagnostic tool over traditional ^18^F‐FDG‐PET for CAYA‐PTC. These findings provide crucial insights into the biological characteristics of CAYA‐PTC patients and offer a foundation for developing personalized therapeutic strategies (Figure S9, Supporting Information).

## 3 Discussion

Intratumoral heterogeneity in both CAYA and adult PTCs presents significant challenges in clinical management.^[^ ^2^ ^]^ This heterogeneity is manifested not only in histopathological diversity but also in genetic and epigenetic alterations, as well as the complex interactions between tumor and its surrounding microenvironment.^[^ ^52^, ^53^ ^]^ However, up to now, there is still a lack of relevant comprehensive and intensive research in CAYA‐PTC patients. In this study, we performed scRNA sequencing to analyze paratumor and localized tumor samples from diverse clinical courses of PTC patients. Overall, our findings significantly enhance the understanding of PTC heterogeneity, providing new dimensions for prognostic stratification and personalized therapies.

One key finding is the identification of CD4+ T follicular helper cell (CD4T_Tfh) and CD8+ T exhaustion (CD8T_Tex). Our investigation into CD4T_Tfh cells within the intricate landscape of tumor‐promoting microenvironments revealed their multifaceted impact on cancer progression.^[^ ^54^, ^55^ ^]^ The dynamic interplay between CD4T_Tfh cells and the TME underscores their potential as key immune responses regulators and presents challenges for therapeutic exploitation. Notably, CD4T_Tfh cells were markedly infiltrated within the CAYA‐PTC microenvironment, particularly in regions associated with enhanced angiogenesis and immune suppression, suggesting their active role in promoting a protumorigenic milieu.^[^ ^27^, ^56^ ^]^ A subset of CD4T_Tfh cells expressed immunosuppressive cytokines, such as *IL‐10* and *TGF‐β*, contributing to an immunosuppressive niche favoring tumor growth.^[^ ^57^, ^58^, ^59^, ^60^ ^]^ The crosstalk between CD4+ Tfh cells and stromal cells within the TME appears critical for tumor progression, highlighting their role in modulating cancer‐associated fibroblasts and endothelial cells, thereby promoting angiogenesis and extracellular matrix remodeling.^[^ ^61^, ^62^ ^]^ CD8T_Tex, characterized by functional impairments and sustained expression of inhibitory receptors, emerges as a central player in tumor‐promoting mechanisms within cancer microenvironments, particularly in CAYA‐PTC patients.^[^ ^63^, ^64^, ^65^, ^66^ ^]^ The exhaustion of CD8T_Tex cells, driven by prolonged antigen exposure and immunosuppressive cues in the TME, resulted in a dysfunctional state marked by upregulated immune checkpoint molecules such as *PD‐1*, *CTLA‐4*, and *LAG3*. These exhausted CD8T_Tex cells, rather than mounting effective antitumor responses, contribute to immune evasion and tumor progression.^[^ ^67^, ^68^ ^]^ Although our study significantly advances the understanding of CD8T_Tex cell exhaustion in tumor promotion, further research is needed to explore the reversibility of exhaustion, the temporal evolution of the microenvironment, and specific therapeutic targets.

We also identified differences in differentiation trajectory of malignant thyrocytes. A recent thyroid cancer analysis confirmed an intermediate state between nontumor bearing healthy tissues and tumor samples.^[^ ^6^, ^69^ ^]^ In our study, we discovered two distinct states (State 2 and State 3) in adult‐PTC, while only one state (State 3) was present in CAYA‐PTC. State 2 possibly represents intermediate transcriptomes with moderate TDS score and lowest fusion score, while the other population represents the lowest TDS score and highest fusion score. The existence of State 2 thyrocytes can be identification of intermediate state from normal tissue to highly aggressive characteristic advanced cancer that genetic alterations of tumor accumulate in a stepwise manner, which mainly manifested as BRAFV600E mutation. This can explain that more than 90% of adult PTC patients in clinical practice mainly manifests as BRAFV600E mutations, and the excellent prognosis.^[^ ^7^, ^70^ ^]^ On the other hand, State 3 thyrocytes presented in both CAYA and adult‐PTC patients as trajectory end that explained CAYA and a proportion of adult patients can exhibit more aggressive clinical manifestations. Thyrocyte_State3 populations, characterized by poor differentiation and high fusion scores, demonstrated enhanced inhibitory signaling with T cells, mediated by immune checkpoint such as *PD‐1*, *CTLA‐4*, and *LAG3*, which were more highly expressed in exhausted T cells in the CAYA cohort. This can indicate that in adults patients, tumors can gradually progress slowly over a long time, while in CAYA, they quickly invade and metastasize outward. Although the universality of the mild thyrocytes needs to be further validated in future studies, these observations and hypotheses, to some extent at least, provide a mechanistic insight into the dynamic tumorigenesis in adult‐PTCs.

The heterogeneity of stromal cells in PTCs has been scarcely characterized. Despite extensive literature supporting a tumor‐promoting role of CAFs, they are now recognized as a heterogeneous entity with complex role in cancer.^[^ ^71^, ^72^ ^]^ Our investigation into the relationships between CAFs, cancer cells, endothelial cells, and immune cells highlights the multifaceted crosstalk within the TME. The interactions among these components orchestrate tumor‐promoting mechanisms, influencing cancer progression and metastasis. We identified four CAF phenotypes, with emCAF_LAMP5 increasing with the malignant of bulk classifications, suggesting a protumorous role. It is generally acknowledged that CAFs confer resistance to anticancer therapies, including chemotherapy and tyrosine kinase inhibitors.^[^ ^73^, ^74^ ^]^ The reciprocal relationship between emCAF_LAMP5 and malignant thyrocyte State 3 is a cornerstone of the tumor microenvironment. Our findings affirm the significant impact of CAFs on the behavior of neighboring cancer cells. These interactions promote immunosuppressive phenotypes, including Treg, Tfh, and Tex, which were more abundant in the CAYA TME. Ligand–receptor analyses highlighted the involvement of ANGPTL4 and VEGFC signaling pathways in these interactions, both of which were significantly upregulated in CAYA patients. These pathways contribute to tumor angiogenesis and lymph node metastasis. Another extremely important finding is the high expression of FAPI in fibroblasts in CAYA‐PTC patients suggesting that ^68^Ga‐FAPI‐PET may offer prominent application potential compared to traditional ^18^F‐FDG‐PET.^[^ ^75^, ^76^, ^77^ ^]^ Nonetheless, this conclusion is derived from a single‐case observation. Multicenter, large‐scale prospective cohort studies with standardized protocols are warranted to rigorously establish its clinical applicability.

Our study underscores the interconnectedness of CAFs, cancer cells, and endothelial cells within the TME. Targeting these intricate interactions may present novel therapeutic avenues, disrupting tumor‐promoting mechanisms and improving cancer treatment strategies. Further elucidation of the molecular and signaling pathways governing these interactions in CAYA‐PTC will be essential for developing targeted strategies to modulate the tumor microenvironment and improve patient outcomes.

In summary, our study comprehensively elucidates and contrasts the ecosystems of CAYA‐PTC and adult‐PTC, suggesting potential diagnostic, prognostic, and therapeutic implications. Our work not only adds depth to the biological understanding of PTC heterogeneity but also provides a benchmark dataset for this malignancy.

## 4 Experimental Section

### Human Specimens

Six paired samples including five CAYA specimens and one adult specimen were collected from patients who had undergone thyroidectomy + central/lateral cervical neck dissection at Shanghai Jiao Tong University School of Medicine Affiliated Renji Hospital and Children's Hospital of Shanghai, Shanghai Jiao Tong University. Postoperative paraffin pathology for all patients was confirmed as papillary thyroid carcinoma by three senior physicians. Information on patients’ demographics, histopathologic tumor type, lymph node metastasis, and performance status were collected from medical records. An 8‐genes panel kit (RigenBio, Shanghai) for gene mutation and gene fusion were performed for all patients. Detection sites included BRAFV600E, TERT promoter region C228T/C250T, KRAS G12C/G12V/Q61R, NRAS Q61R, HRAS Q61R, CCDC6 (E1/8)‐RET (E12) (RET/PTC1), PAX8 (E10)‐PPARG (E2), and ETV6 (E4)‐NTRK3 (E14). Protocols for investigations involving human tissues used in this project were approved by the Ethics Committee of Renji Hospital, School of Medicine, Shanghai Jiaotong University (050432‐4‐1805C).

### Tumor Dissociation Frozen

Tissue samples are stored in MACS Tissue Storage Solution (Miltenyi Biotec) immediately after surgically removed. The tissues were removed and stored in until processing. Tissue samples were processed as described below. Briefly, the tissues were taken in sterile petri dishes and cleaned with DPBS precooled at 4 °C. Then, use sterile scissors and tweezers to cut the tissue into small pieces below 1 mm^3^ on ice. Collagenase Type II (Sigma‐Aldrich, Shanghai) was added and transferred to 15 mL centrifuge tube, vibrated in 37 °C water bath for 30 min. The cell suspensions were filtered using 70 µm nylon meshes filter, resuspended in DPBS and then appropriate amount of RBC lysis buffer (Beyotime, Shanghai) were added to remove erythrocytes. Stained using 0.4% trypan blue was mixed well and then it was placed to the Countess II Automated Cell Counter (Thermos Fisher Scientific, USA) for cell count and cell viability measurements.

### Single‐Cell Sequencing, Quality Control, and Batch Effect Handling

Cells suspensions (300–600 living cells per microliter determined by Count Star) were washed in PBS with 0.04% BSA and resuspended before loading cells onto the Chromium single cell controller (10× Genomics) to capture single cells in droplets according to the manufacturer's protocol. Libraries were prepared using Single Cell 3′ Library Gel Bead Kit V2 (10× Genomics). Finally, sequencing was performed on the Illumina Novaseq 6000 sequencer with a sequencing depth of at least 132 920 reads per cell and 150 bp (PE150) paired‐end reads (performed by NovelBio, Shanghai). To remove low‐quality cells and technical artifacts, DoubletFinder (version 2.0.6) was employed to calculate pANN scores and subsequently excluded the top X% of cells exhibiting the highest doublet probability based on this metric. The following quality control steps were applied to the scRNA‐seq dataset. i) The unique molecular identifiers (UMIs) count per spot is below 500 were not considered (nUMI <500). ii) Cells that had fewer than 250 expressed genes (nGene < 250). iii) Lower novelty score (log10GenesPerUMI < 0.80) and iv) over 20% of UMIs derived from the mitochondrial genome were removed (mitoRatio > 20). A total of 143 477 high‐quality cells were retained. After quality control and filtering, the feature‐barcode matrices of each library were processed by Seurat for normalization, dimension reduction, batch effect removal, graph‐based clustering, cluster‐specific marker gene detection, and visualization. Data were processed using Seurat (version 4.3.0) NormalizeData was first performed followed by identification of highly variable genes using FindVariableFeatures. Batch correction was then conducted using canonical correlation analysis via FindIntegrationAnchors and IntegrateData. Principal component analysis was performed on the integrated dataset. Integration quality was assessed by the average silhouette width (ASW) calculated from 10 000 randomly sampled cells (ASW = 0.15), indicating minimal batch effects (Figure S1, Supporting Information). Leiden clustering was applied, and cluster‐specific markers were identified by Wilcoxon rank‐sum test. Cell distributions were visualized using UMAP and *t*‐distributed stochastic neighbor embedding.

### Gene Enrichment Analysis

The “clusterProfiler” package was employed for enrichment analysis and utilized the msigdbr package to extract signature gene sets from molecular signature databases such as KEGG, Gene Ontology (GO), and Hallmark. Initially, differentially expressed genes were ranked based on log fold change (logFC) and adjusted *P*‐values. These genes were then inputted into gene set enrichment analyses as gene sets. Gene Set Enrichment Analysis was applied and selected gene was set to identify biological pathways that were upregulated across different cell subpopulations. Additionally, to display cell subpopulation‐specific signaling pathway activity in heatmaps, the average gene expression level was calculated for each cell subpopulation using GSVA (version 1.30.0). For the scores shown in violin plots, the activity of each gene set by calculating the average expression of genes within the set (after *z*‐score transformation) was measured.

### Tissue Distribution of Clusters

The Ro/e across different tissues was calculated to quantify the tissue preference of each cluster. Ro/e was defined as the ratio of *N*_observe_/*N*_expect_, where *N*_observe_ represents the number of cells in a cluster originating from a particular tissue, and *N*_expect_ is the expected number of cells, calculated using a chi‐squared test. A cluster was considered enriched in a specific tissue if Ro/e > 1. For most clusters, tissue preference was classified using the following Ro/e index: (+++, Ro/e > 3; ++, 1 < Ro/e ≤ 3; +, 0.2 ≤ Ro/e ≤ 1; +/−, 0 < Ro/e < 0.2; and −, Ro/e = 0).

### Cell Score

To evaluate the expression activity of specific predefined gene sets in single cells, a cell scoring methodology was employed. The essence of this approach is to compute the average expression enhancement of a predefined gene set relative to a background gene set within individual cells. Specifically, for a given cell *i* and gene set *j* (denoted as *Gj*​), the cell score *SCj*​(*i*) is defined by quantifying the difference in expression of *Gj*​ in cell *i* compared to the average expression of a control gene set (*Gjcont*​). The control gene set is randomly selected based on overall expression levels to ensure its similarity to the target gene set in terms of expression level and size distribution. The calculation formula is: *SCj*​(*i*) = mean(*Er*​(*Gj*​,*i*))−mean(*Er*​(*Gjcont*​,*i*)). Here, *Er*​ represents the relative expression, that is, the expression of each gene in *Gj*​ within cell *i* relative to the average expression of *Gjcont*​. This calculation method is implemented through the “AddModuleScore” function in the Seurat package, executed under default parameter settings.

### Developmental Trajectory Inference

The Monocle 3 R package (version 2.10.1) was used to infer the developmental trajectories of T cells. By employing the “DifferentialGeneTest” function, differentially expressed genes were identified between clusters, selecting the top 2000 genes with a *q*‐value < 0.01. Based on these genes, cluster‐based expression trajectories were constructed. Furthermore, leveraging the analysis results from Seurat, Monocle 2 was employed to construct single‐cell trajectories of EMT. In the selection of genes for analysis, the following criteria were established: 1) expression in at least four cells, 2) an average expression level greater than 0.1, and 3) the *q*‐value <0.01 in the differential gene expression analysis. Subsequently, the “plot_pseudotime_heatmap” function was further utilized in Monocle 2 to explore the pivotal roles of series of genes during cell differentiation. This step allowed to meticulously observe and analyze the patterns of gene expression changes over pseudotime.

### Developmental Potential Inference

CytoTRACE software (version 0.3.3), a tool for evaluating cellular developmental potential based on single‐cell RNA sequencing (scRNA‐seq) data, was employed. CytoTRACE predicts by analyzing the number of genes expressed in each cell, with its core principle being the negative correlation between the number of expressed genes and transcriptional diversity within a cell. In essence, the fewer genes a cell expresses, the greater its developmental potential tends to be, as less differentiated cells tend to express fewer genes, while more mature cells exhibit higher transcriptional diversity. CytoTRACE was utilized to infer the potential developmental trajectories of thyroid epithelial cells.

### TF Regulatory Network Analysis

The SCENIC package (version 1.2.4) was used to construct transcription factor (TF) regulatory networks in scRNA‐seq data, identifying cellular functional states. The quantified matrix of TF activity was imported into Seurat to identify subgroup specific TFs via “FindAllMarkers” function. Clustering was based on the Regulon‐specific CSI, a metric used to assess the relevance and specificity of a particular Regulon across different cell subpopulations, thereby identifying TFs that may play key roles in cellular state transitions and functional specialization.

### Cell–Cell Interactions

Cell–cell interaction analyses were conducted using the CellPhoneDB (Python package, version 2.1.4) to predict cell–cell communication networks, which integrates a publicly available L–R pair database and a statistical framework. The interaction networks were explored between all cell subpopulations and the interaction strengths among them were presented in heatmaps. Communication events between different cell types were displayed in the form of connecting lines. For analyzing intercellular communication within the TME, the CellChat algorithm (version 2.1.1) was utilized to infer interactions among cells and to identify differential interactions between CAYA and Adult samples. Following the official workflow, the “createCellChat” function was used to import gene expression data into CellChat. Functions such as “identifyOverExpressedGenes,” “identifyOverexpressedInteractions,” and “projectData” were primarily employed to detect significant cell–cell interactions among the cells. The “compareInteractions” and “RankNet” functions were used for comparative interaction analysis between different groups of samples. The “netAnalysis_signalingRole_scatter” function calculated the input and output interaction strengths across datasets.

### Transcriptome Sequencing Analysis: TCGA Data Analysis

Gene expression data were downloaded using the Bioconductor TCGA biolinks package (version 2.14.0), and the clinical data were downloaded from the Genomic Data Commons Data Portal (https://gdc‐portal.nci.nih.gov/). The count data were converted to transcripts per million (TPM) using the “countToTpm_matrix” function in the R package “GeoTcgaData”. All TCGA data used in this study are derived from adult PTC patients, as pediatric cases are not included in the TCGA dataset. The correlations between marker genes expression and the relative abundance of different subclusters were calculated using the Spearman method. The correlation of each marker gene with OS and PFS was calculated using a Cox proportional hazards model (R Package survival, version 3.2‐7). Kaplan–Meier survival curves were plotted to show the differences in of survival curves between the high and low expression group.

### Bulk Whole Transcriptome Sequencing and Data Processing

With signed informed consents, surgically resected tissue samples were collected from individuals of forty CAYA and ten adult PTC at authors’ institutes. Total RNA was isolated from frozen tissue sections, each containing ≈50–100 mg of tissue, utilizing TRIzol Reagent (Life Technologies, Carlsbad, CA, USA) in accordance with the manufacturer's protocol. The quality, quantity, and integrity of the extracted RNA were assessed using a SmartSpec Plus spectrophotometer (Bio‐Rad, Hercules, CA, USA). Subsequently, whole transcriptome libraries were prepared and subjected to deep sequencing by Sangon Biotech Co., Ltd. (Shanghai, China), utilizing the Illumina HiSeq 2000 platform (San Diego, CA, USA) for sequencing. The sequencing data quality was evaluated via FastQC (version 0.11.2). Alignment of reads to the human genome (hg38) was performed using HISAT2 (version 2.1.0) and RSeQC (version 2.6.1). Genes exhibiting low expression were excluded based on a maximum read count threshold of <20 across all samples. Bcftools (version 1.5) facilitated gene mutation analysis within each sample, whereas SnpEff (version 2.36) determined the genomic structure variation site distribution, applying filters for quality scores >20 and coverage >8. Gene expression quantification in each sample was normalized using the TPM algorithm.

### Digital Cytometry Using CIBERSORTx

To assess the relative abundance of each cell type identified in single‐cell sequencing, genes with a differential analysis fold change >2 within cell subpopulations from single‐cell sequencing were used as subpopulation marker genes. The average TPM levels of these marker genes were log_2_ transformed and used as a reference. CIBERSORTx was executed with default parameters to identify the abundance of cell subpopulations in both TCGA and transcriptome sequencing datasets.

### Survival Analysis

Using CIBERSORTx absolute scores, the percentage of each cell subpopulation present in each sample was calculated, and these values were then utilized as continuous covariates in a Cox proportional hazards regression model (CoxPH) for OS and PFS analysis. For categorical analysis, the TCGA‐THCA cohort served as the testing dataset, with the optimal binary classification threshold determined using the “survival” (version 3.5.7) and “survminer” (version 0.4.9) R packages. Statistical significance was assessed using the log‐rank test (univariate analysis) or the CoxPH regression model (multivariate analysis).

### Multiplex Immunohistochemistry (MxIHC)

Multiplex IHC staining of formalin‐fixed paraffin‐embedded tissues was conducted using the bry‐0067‐050 (Runnerbio, Shanghai) in strict adherence to the manufacturer's protocols. Expression of *LAMP5* was used to identify emCAF_LAMP5, *CD36* to identify lpmCAF_CD36, *FBLN1* to identify iCAF _CFD, and *MYH11* (*SMMHC*) to identify myoCAF_MYH11 in Figure 5D. *ANGPTL4*, *LAMP5*, *CD31*, and *PANCK* were selected as optimal IHC markers for emCAF, lymphatic EC, and thyrocyte, respectively, for Figure 7G. A sequential application of antibodies against *ANGPTL4* (NBP2‐80039, NOVUS, USA), *LAMP5* (NBP1‐84246, NOVUS, USA), *CD31* (BD, USA), *CD36* (18836‐1‐AP, Proteintech, China), *FBLN1* (NBP1‐84725, NOVUS, USA), *MYH11* (21404‐1‐AP, Proteintech, China), *VEGFC* (22601‐1‐AP, Proteintech, China), *VEGFR3* (20712‐1‐AP, Proteintech, China), and Pan‐CK (BD, USA) was performed, followed by incubation with horseradish peroxidase‐conjugated secondary antibodies and subsequent tyramide signal amplification. Post‐tyramide signal amplification, slides underwent microwave heat treatment. Upon labeling of all aforementioned antigens, nuclei staining was achieved using DAPI. These stained slides were then subject to scanning with the PANNORAMIC SCAN II to acquire multispectral images. This system is designed to capture the fluorescent spectra across a range of 20 nm wavelength intervals, spanning from 420 to 720 nm, with uniform exposure durations. For each slide, five fields within tumor areas enriched in immune cells were meticulously selected for imaging. These selected fields were scanned to secure multispectral images, employing the PANNORAMIC SCAN II for the capture of fluorescent spectra across the same 20 nm wavelength intervals, ensuring consistent exposure times.

### Immunohistochemistry Staining

Surgical specimens from PTC patients were fixed in 4% paraformaldehyde and subsequently embedded in paraffin. Sections underwent deparaffinization followed by antigen retrieval via heat induction in 10 mm citrate buffer for 5 min. The sections were then treated with 3% hydrogen peroxide for 30 min and blocked using AquaBlock (PP82, EastCoast Bio) for 1 h at ambient temperature. For tissue staining, primary antibodies targeting FAP (S0B2166, STARTER, China) and a goat anti‐rabbit/mouse IgG secondary antibody were utilized. Visualization of sections was achieved using an Olympus microscope, equipped with an Olympus camera and Vision 4.1 software, at magnifications of 200× and 400× for each sample. Quantification of immunohistochemistry (IHC) and automated scoring in the study were performed using ImageJ (version 2.1.0) and the open‐source plugin “IHC Profile”. IHC images were categorized based on cytoplasmic or nuclear predominance in staining and processed in the software as per the guidelines. Scoring was designated as high positive (3+), positive (2+), low positive (1+), and negative (0). Blinding was maintained for the origin of samples, whether from CAYA or adult patients, to the experimenters and researchers interpreting the staining outcomes.

### Flow Cytometry

Briefly, tumor and paratumor tissues were cut into small pieces below 1 mm3 using sterile scissors and tweezers and digested at 37 °C for 15 min with 1 mg mL^−1^ Collagenase IV and 0.1 mg mL^−1^ DNase I (Roche) on rotary shaker. Digestion was stopped by RPMI‐1640 (with 5% fetal bovine serum (FBS)) and cells were filtrated through 70 µm cell strainers and washed twice with 2 mL PBS. Cells were resuspended in the staining buffer and stained with following antibodies on ice for 30 min: anti‐*CD45* (563204, BD Pharmingen, USA), anti‐*CD11B* (557396, BD Pharmingen, USA), anti‐*CD68* (564943, BD Pharmingen, USA), anti‐*CD86* (555660, BD Pharmingen, USA), anti‐*CD206* (555954, BD Pharmingen, USA), anti‐*CD3* (557943, BD Pharmingen, USA), anti‐*CD19* (561742, BD Pharmingen, USA), anti‐*CD4* (566320, BD Pharmingen, USA), anti‐*CD8* (555635, BD Pharmingen, USA), anti‐*CD25* (562443, BD Pharmingen, USA), anti‐*CD127* (560822, BD Pharmingen, USA), anti‐*CD14* (555397, BD Pharmingen, USA), anti‐*HLA‐DR* (555812, BD Pharmingen, USA), anti‐*CD11C* (562561, BD Pharmingen, USA), anti‐*CD123* (17‐1239‐42, BD Pharmingen, USA), anti‐*CD56* (566400, BD Pharmingen, USA), and anti‐*CD16* (563690, BD Pharmingen, USA). Prior to flow cytometry, DAPI was added to cell suspension (Sigma #D8417, 1 µg mL^−1^). The signal was detected via the PE channel. Cells were maintained at 4 °C and analyzed on Cytek Aurora flow cytometer (Cytek Biosciences). Data were collected in SpectroFlo (v.3.0.0) and analyzed in FlowJo (v.10.6.2).

### Cell Culturing and Reagents

BCPAP cells were cultured in RPMI‐1640 medium supplemented with 10% FBS (Invitrogen‐Gibco), 100 U mL^−1^ penicillin G sodium salt, and 100 U mL^−1^ streptomycin sulfate (Invitrogen‐Gibco). The cells were incubated at 37 °C in a 5% CO_2_ atmosphere. Human recombinant TGF‐β1 was sourced from R&D Systems (Minneapolis, MN, USA).

### Western Blot

Total protein was extracted using RIPA buffer (Sigma), separated by 12% SDS‐PAGE, and transferred to PVDF membranes. After blocking with 5% nonfat milk, the membranes were incubated overnight at 4 °C with primary antibodies against Claudin‐1 (28674‐1‐AP), N‐cadherin (66219‐1‐Ig), vimentin (60330‐1‐Ig), β‐catenin (66379‐1‐Ig), and GAPDH (60004‐1‐Ig). The membranes were then incubated with the corresponding secondary antibodies at room temperature for 2 h. All antibodies were purchased from Santa Cruz Biotechnology (Santa Cruz, CA, USA). Protein bands were visualized using an ECL Western Blot Kit (CWBIO).

### Statistical Analysis

All statistical analyses were performed using R software (version 4.3.1). Continuous variables are expressed as mean ± standard deviation (SD) and compared using two‐tailed unpaired Student's *t*‐tests after verifying normality assumptions. Categorical variables are reported as counts (percentages) and analyzed by chi test or Fisher's exact test, as appropriate. Bivariate associations were examined using Pearson correlation coefficients. Survival outcomes were analyzed using Kaplan–Meier methodology, with between‐group differences evaluated by log‐rank test. Independent prognostic factors were identified through multivariable Cox proportional hazards regression modeling. Data presented reflect findings from a minimum of three biologically independent experimental replicates. A two‐sided *P* level of 0.05 was established a priori for determining statistical significance.

### Ethical Statement

The studies involving human participants were reviewed and approved by Clinical Research Ethics Committee of Renji Hospital, School of Medicine, Shanghai Jiaotong University. The patients/participants provided their written informed consent to participate in this study.

## Conflict of Interest

The authors declare no conflict of interest.

## Author Contributions

K.G., L.Y. and Z.H. contributed equally to this work. Z.B.L, J.B.L., K.R.D., and Z.Y.W. designed the study. Y.S., M.J.F., Z.X.C., M.Q., and Z.C. collected and prepared patient tissues. L.Y.Y., Z.R.H., Y.S., Y.F., and A.Q.L. performed the experiments. K.G., L.Y.Y., Z.R.H., A.Q.L., and K.Q. did the statistical analyses. K.G., L.Y.Y., Z.R.H., and L.L.T. prepared figures. K.G., L.Y.Y., and Z.R.H. reviewed the results, interpreted data, and wrote the manuscript. All authors have made an intellectual contribution to the manuscript and approved the submission.

## Supporting information



Supporting Information

Supplemental Figure 1

Supplemental Figure 2

Supplemental Figure 3

Supplemental Figure 4

Supplemental Figure 5

Supplemental Figure 6

Supplemental Figure 7

Supplemental Figure 8

Supplemental Figure 9

Supporting Tables

Supplemental Data

Supplemental Data

Supplemental Data

Supplemental Data

## Data Availability

The scRNA‐seq generated during this study are available at the NCBI (National Center for Biotechnology Information) under accession number GSE281736 that can be accessed at https://www.ncbi.nlm.nih.gov/geo/query/acc.cgi?acc=GSE281736. The adult patients’ single‐cell RNA‐seq data from Pu et al. were downloaded from the Gene Expression Omnibus (GEO) database under accession number GSE184362. All other data are available in the main text or in the Supporting Information.
