# Correction to “Single‐Cell RNA Sequencing Reveals the Heterogeneity in Differentiation Trajectory and Tumor Microenvironment Leading to More Aggressive Phenotypes of Papillary Thyroid Cancer in Children and Young Adult Patients”

**DOI:** 10.1002/advs.77252

**Published:** 2026-08-21

**Authors:** Kai Guo, Lingyi Yang, Zirui Huang, Kai Qian, Yuan Shi, Mengjia Fei, Yuan Feng, Zixian Chen, Ming Qiu, Linglin Tang, Anqi Li, Zhibao Lv, Jiangbin Liu, Zhou Chen, Kuiran Dong, Zhuoying Wang

**Affiliations:** ^1^ Department of Head and Neck Surgery Renji Hospital School of Medicine Shanghai Jiaotong University Shanghai China; ^2^ Department of Nuclear Medicine Renji Hospital School of Medicine Shanghai Jiaotong University Shanghai China; ^3^ Department of Pathology Ruijin Hospital School of Medicine Shanghai Jiaotong University Shanghai China; ^4^ Department of General Surgery Children's Hospital of Shanghai Shanghai Jiao Tong University Shanghai China; ^5^ Department of Pediatric Surgery Children's Hospital of Fudan University Shanghai China

https://doi.org/10.1002/advs.202417672

In the originally published article, the ethics approval information was incomplete and requires clarification.

On page 17, under section “4. Experimental Section,” the following text was incorrect:

“Protocols for investigations involving human tissues used in this project were approved by the Ethics Committee of Renji Hospital, School of Medicine, Shanghai Jiaotong University (050432‐4‐1805C).”

The correct statement should read:

“Protocols for investigations involving human tissues reported in this article were reviewed and approved by the Ethics Committee of Fudan University Shanghai Cancer Center (050432‐4‐1805C). Following transfer of the research protocol to Renji Hospital, Shanghai Jiao Tong University School of Medicine, the continuing research protocol was reviewed and approved by the Ethics Committee of Renji Hospital, Shanghai Jiao Tong University School of Medicine (KY2025‐146‐C).”

On page 19, under the section “Ethical Statement,” the following text was incorrect:

“The studies involving human participants were reviewed and approved by the Clinical Research Ethics Committee of Renji Hospital, School of Medicine, Shanghai Jiaotong University.”

The correct statement should read:

“The studies involving human participants reported in this article were reviewed and approved by the Ethics Committee of Fudan University Shanghai Cancer Center. Following transfer of the research protocol, the continuing research protocol was reviewed and approved by the Ethics Committee of Renji Hospital, Shanghai Jiao Tong University School of Medicine.”

These corrections clarify the ethics approval information and do not affect the results, interpretation, or conclusions of the article.

The authors apologize for any inconvenience caused.

