# Supplementary material for: Single‐Cell RNA Sequencing Reveals the Heterogeneity in Differentiation Trajectory and Tumor Microenvironment Leading to More Aggressive Phenotypes of Papillary Thyroid Cancer in Children and Young Adult Patients
Source: Adv Sci (Weinh). 2025 Jul 28;12(39):e17672. doi: 10.1002/advs.202417672 (PMC12533407; doi:10.1002/advs.202417672)
Supplement: Supplementary file 1 — Supporting Information [file ADVS-12-e17672-s005.docx]

**Figure S1. A single cell atlas of PTC by Garnett.** A. PTC atlas marker quality plot, x axis represents the ambiguity score, color correspond to the percentage of nominated cells for a given cell type. B. UMAP with Garnett-based cell-type annotation. The legend on the right demonstrated that the 9 colors represent different cell types. C. UMAP visualization of scRNA before (left) and after (right) batch effect correction. The legend below explains that the different colors represent the origins of the sequencing samples (T: Tumor tissue; P: Paratumor tissue). D. Frequences of each subcluster divided by different groups (CAYA: children and young adults; HT: cowith-Hashimoto's thyroiditis; NHT: with no Hashimoto's thyroiditis; N0: with no cervical lymphoid metastasis; N1: with cervical lymphoid metastasis). E. The comparison of immune cell, thyrocytes, endothelial cells, fibroblasts and STMN1_cells from different groups. Significance *P* value was determined by *chi*-squared test.

**Figure S2. The different ratio of CD4+T cells, Treg and CD8+T cells between CAYA and adult PTC patients measured by flow cytometry.** A. Flow cytometry schemes of tissue samples. CD45+cells were first selected from single live lymphocytes. Representative scattered figures of CD3+CD4+T cells (B), CD25+CD127-Treg (C) and CD3+CD8+T cells (D) in CAYA (n=10) and adults (n=10).

**Figure S3. The supplemental figure related to CD4+T and CD8+T cells. A.** Ucell mapping of key markers for CD8+T cell subclusters. Color gradients in the right bottom legend reflect the magnitude of signature enrichment, with higher scores indicating stronger expression. B. Violin plot indicating the expression of exhaustion related genes (*PDCD1*, *CTLA4*, *LAG3*, *TIGIT*) in 10 subclusters of CD8+T cells. C. Multiplex immunofluorescence staining showed *PDCD1* and *CTLA4* of CD3+ +T cells existed in CAYA (n=3) and adult PTC tissues (n=3).

**Figure S4. Characteristics of NK/B cells and effects on the tumor ecosystem.** A. UMAP of NK cells, with split by group (top right) and distribution of tissues (bottom right), and Ucell mapping of key markers for NK cell subclusters (bottom). Color gradients in the bottom legend reflect the magnitude of signature enrichment, with higher scores indicating stronger expression. B. Heatmap showing the expression of marker genes in each subtype of NK cells. C. Tissue preference of each NK cell cluster estimated by the ratio of observed to expected cell numbers (Ro/e). D. Violin plot showing the cytotoxicity score in each NK subcluster. E. Violin plot showing the activation score in each NK subcluster. F. UMAP of B cells, with split by group (top right) and distribution of tissues (bottom right), and Ucell mapping of key markers for NK cell subclusters (bottom). G. Heatmap showing the expression of marker genes in each subtype of B cells. H. Tissue preference of each B cell cluster estimated by the ratio of observed to expected cell numbers (Ro/e). I. Violin plot showing the Bcell receptor score in each B cell subcluster and separated by CAYA and adult. *****P* < 0.0001; two-sided *t*-test. J. Significantly identified pathway that enrich in CAYA versus adult PTC by KEGG pathway analysis. Normalized enrichment score (NES), corrected for multiple comparisons using false discovery rate (FDR) method.

**Figure S5. Characteristics of myeloid and endothelial cells and their effects on the tumor ecosystem.** A. UMAP of myeloid cells, with split by group (top right) and distribution of tissues (bottom right). TAM: Tumor-associated macrophage, Mono: monocytes, DC: Dendritic Cell, pDC: plasmacytoid Dendritic cell. B. Heatmap showing the expression of marker genes in each subtype of myeloid cells. C. Tissue preference of each myeloid cell cluster estimated by the ratio of observed to expected cell numbers (Ro/e). D. Ucell mapping of key markers for myeloid cell subclusters. Color gradients in the bottom of each cluster reflect the magnitude of signature enrichment, with higher scores indicating stronger expression. E-F. Violin plot showing the M2 score(E) and phagocytosis score(F) in each myeloid cell subcluster (left) and separated by CAYA and adult (right). **P* < 0.05, ***P* < 0.01, ****P* < 0.001, *****P* < 0.0001; two-sided *t*-test. H. UMAP of endothelial cells, with split by group (top right) and distribution of tissues (bottom right). H. Heatmap showing the expression of marker genes in each subtype of endothelial cells. I. Tissue preference of each endothelial cell cluster estimated by the ratio of observed to expected cell numbers (Ro/e). J. Ucell mapping of key markers for myeloid cell subclusters.

**Figure S6.** **The different ratio of NK/B/Myeloid cells between CAYA and adult PTC patients measured by flow cytometry.** Representative scattered figures of CD56+ NK, CD16+ NK, CD19+B cells, CD86+CD206-M1, CD206+M2 cells and HLA-DR+CD123+pDC, HLA-DR+CD11C+cDC in CAYA (n=10) and adults (n=10).

**Figure S7. Gene expression, tissue distribution and EMT analysis in different groups.** A. TAGLN and RGS5 expression in CAF subclusters (cluster 0-3). B. Heatmap showing the most significantly upregulated Hallmark pathways in each subcluster and different tissues calculated through GSVA, colored by z transformed mean GSVA scores. C. Frequency of CAF subclusters in different tissues. **P* < 0.05, ***P* < 0.01, ns: not significant; two-sided *t*-test. D. Proportion of CAF subclusters from different samples and groups. E. Tissue preference of each CAF subcluster estimated by the ratio of observed to expected cell numbers (Ro/e). F. Multiplex immunofluorescence staining showed major CAF clusters existed in CAYA (n=63) and adult (n=80) normal tissues. G. The violin plot showed the different percentage of positive cells for markers used in mxIHC separated by groups. **P* < 0.05, ***P* < 0.01, ****P* < 0.001, *****P* < 0.0001; two-sided *t*-test. (CAYA: n=78, Adult: n=80) H. The differentially expressed genes (rows) along the pseudotime (columns) were hierarchically clustered into four subclusters. The representative annotated pathways of each subcluster are provided. I. EMT score in four CAF subclusters (Left) and in different group (right). **P* < 0.05, ***P* < 0.01, ****P* < 0.001, *****P* < 0.0001; two-sided *t*-test. J. BCPAP cell morphology changes when pretreated with TGF-b1 (5ng/mL) in the time of 0h, 9h and 24h (n=3). K. The protein levels of N-cadherin, vimentin, B-cadherin and Claudin-1 in BCPAP cells were detected by western blot (n=3).

**Figure S8. Communication among tumor microenvironment components.** A-C. Heatmap showing interactions between all subgroups in total samples (A) or CAYA (B) or adult (C) by CellphoneDB. D-F. Heatmap showing the strength of incoming and outgoing events in interactions between different clusters in the COLLAGEN signaling pathway (D), FN1 signaling pathway (E) and PERIOSTIN signaling pathway (F). Histograms separately count the overall intensity of outgoing (y axis) and incoming (x axis) events for each cluster.

**Figure S9. Diagram illustrating the reprogramming of TME in PTC progression.** Top, schematic diagram of the TME and cell-cell interactions in primary PTC. Bottom, schematic diagram of the TME and cell-cell interactions in CAYA-PTC (left) and adult-PTC (right).
