# Supplementary figures and images for: Single‐Cell RNA Sequencing Reveals the Heterogeneity in Differentiation Trajectory and Tumor Microenvironment Leading to More Aggressive Phenotypes of Papillary Thyroid Cancer in Children and Young Adult Patients
Source: Adv Sci (Weinh). 2025 Jul 28;12(39):e17672. doi: 10.1002/advs.202417672 (PMC12533407; doi:10.1002/advs.202417672)

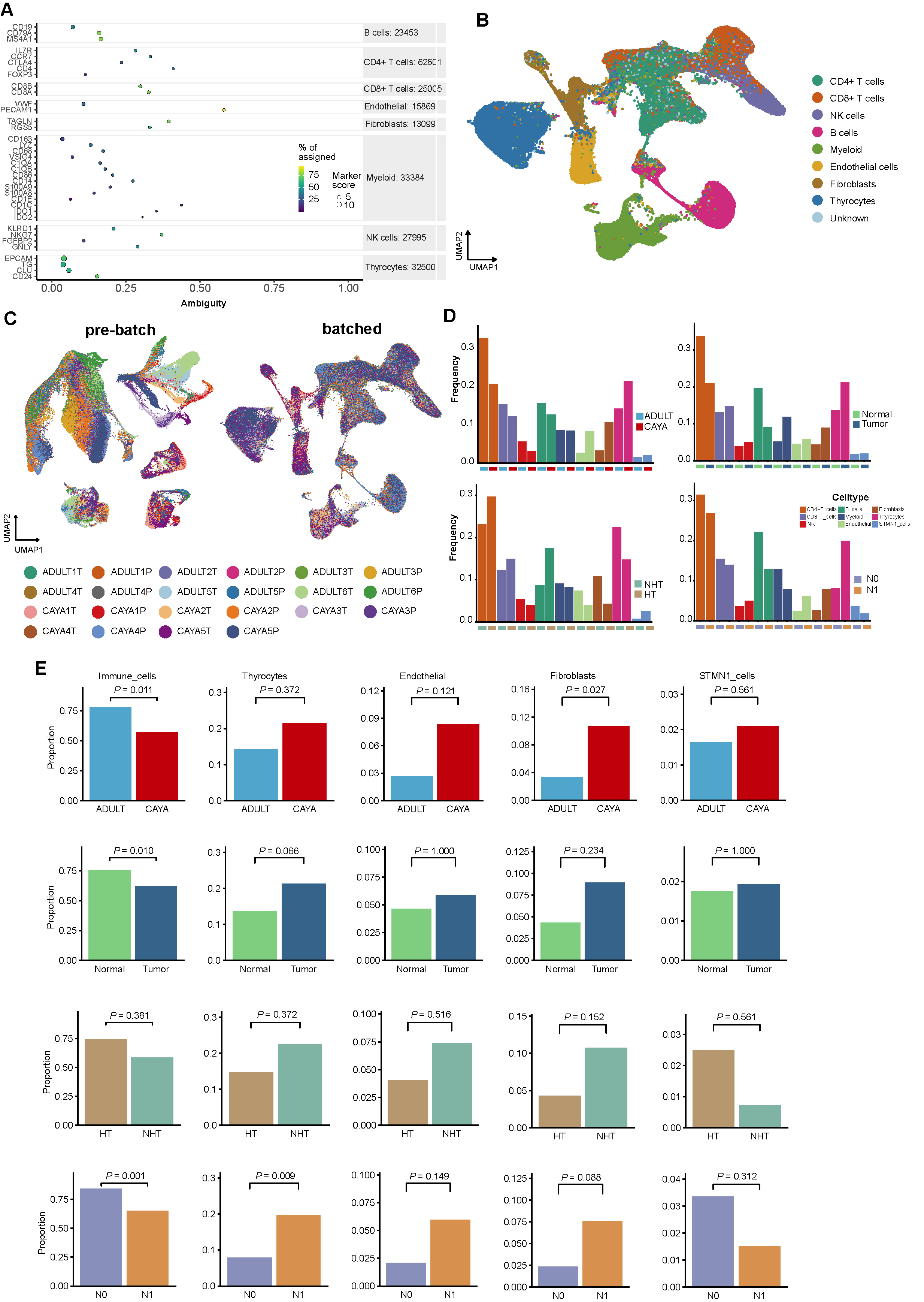

Supplement: Supplementary file 2 — Supplemental Figure 1 [file ADVS-12-e17672-s006.jpg]

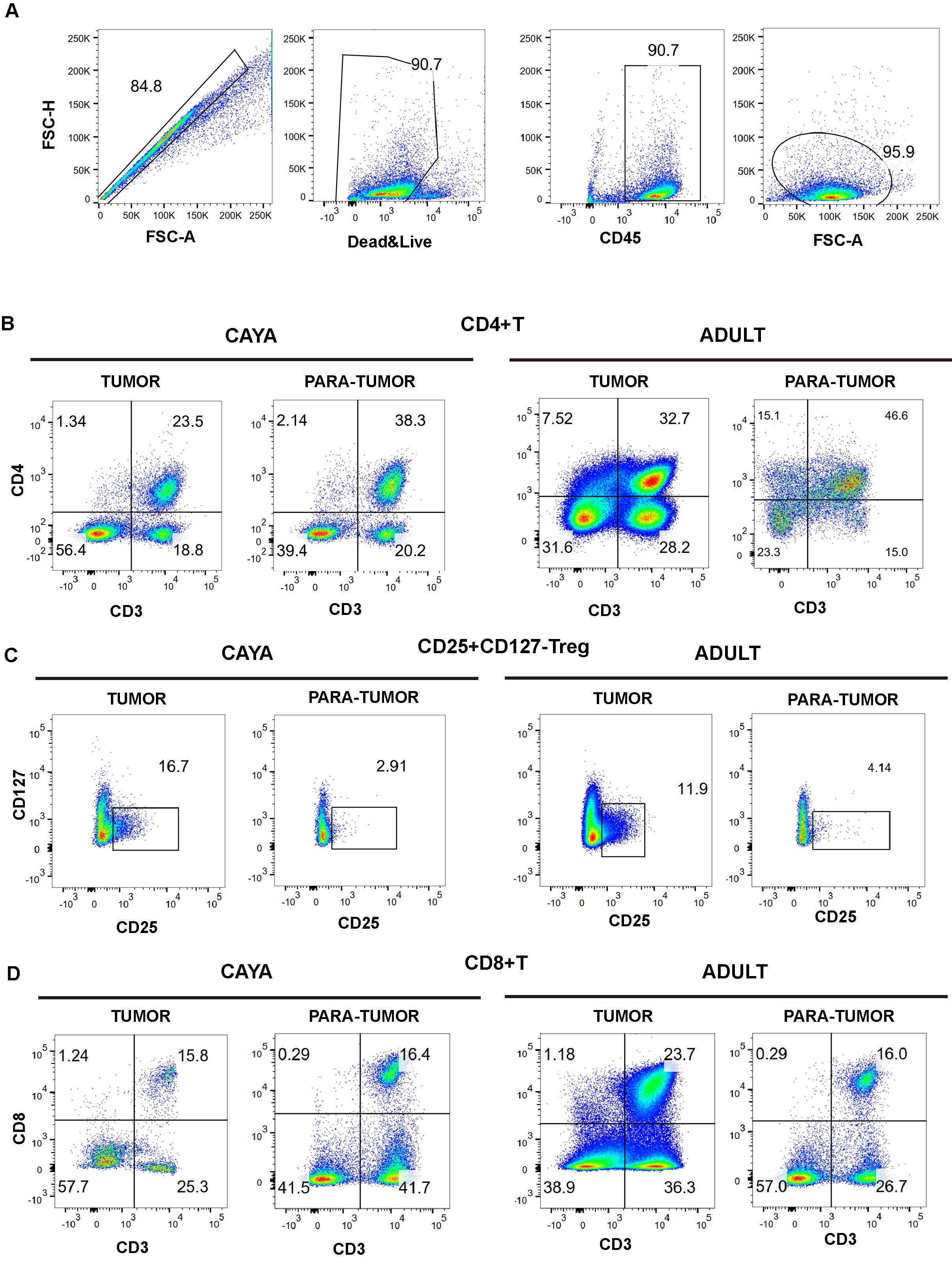

Supplement: Supplementary file 3 — Supplemental Figure 2 [file ADVS-12-e17672-s011.jpg]

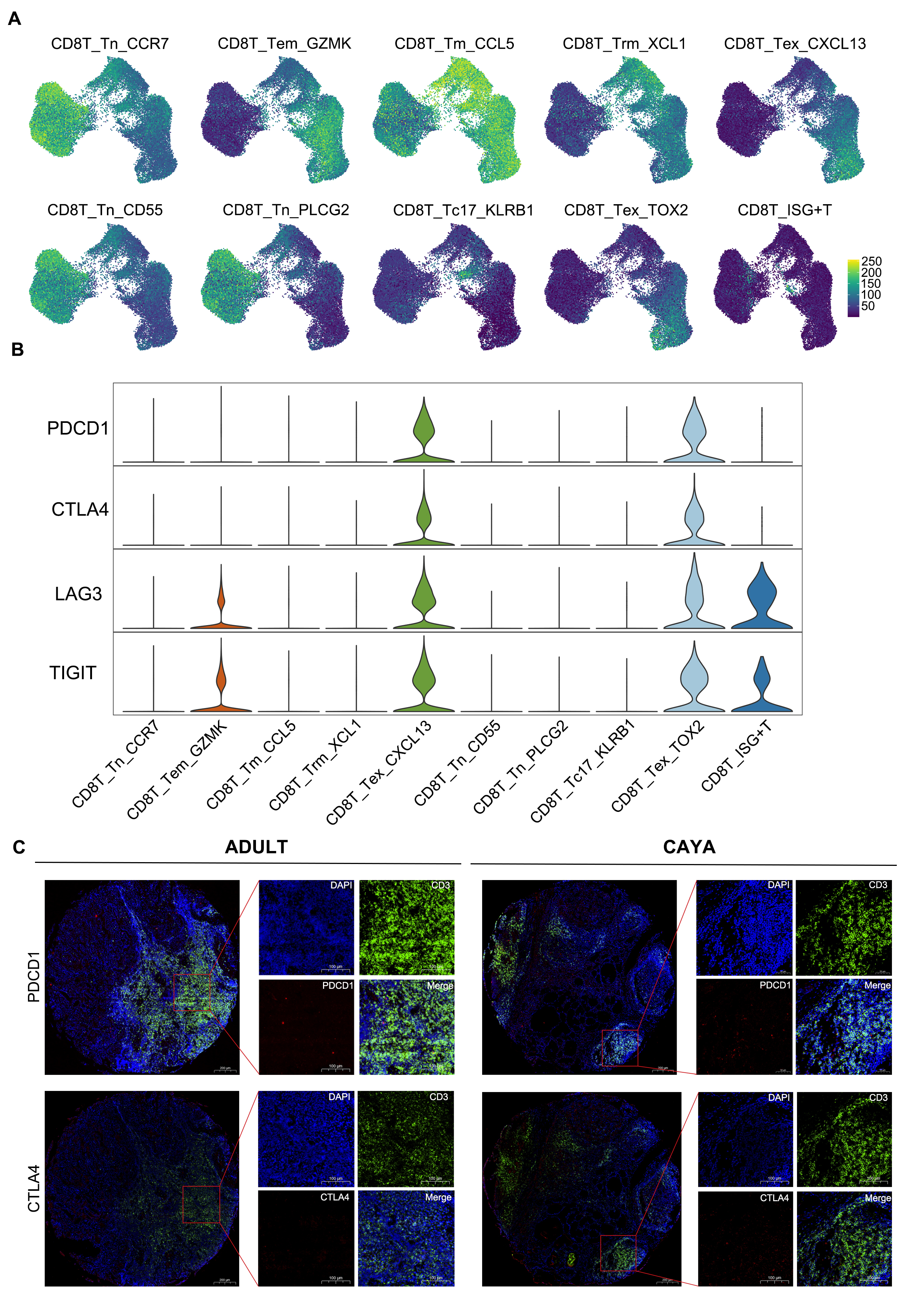

Supplement: Supplementary file 4 — Supplemental Figure 3 [file ADVS-12-e17672-s004.jpg]

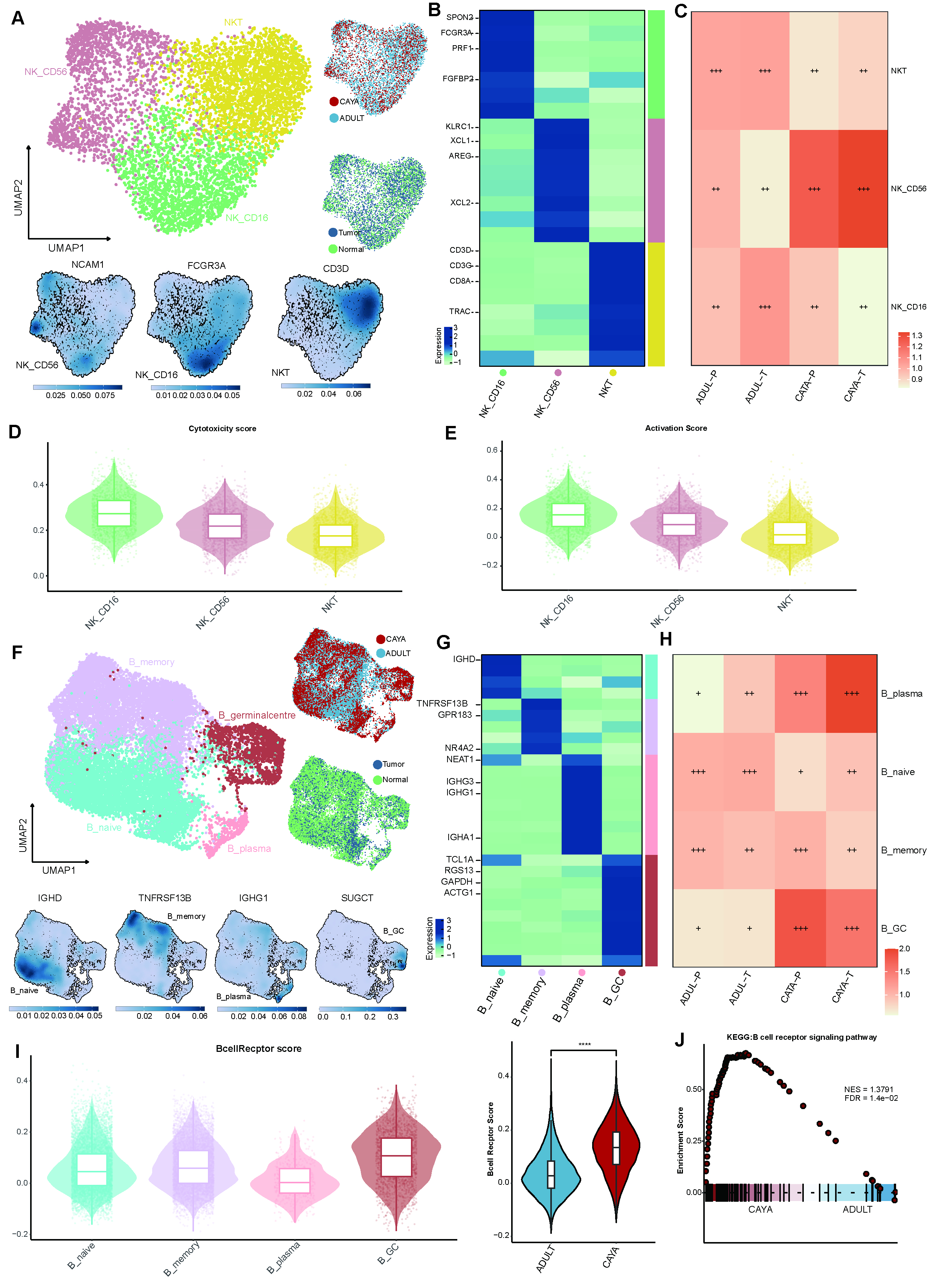

Supplement: Supplementary file 5 — Supplemental Figure 4 [file ADVS-12-e17672-s012.jpg]

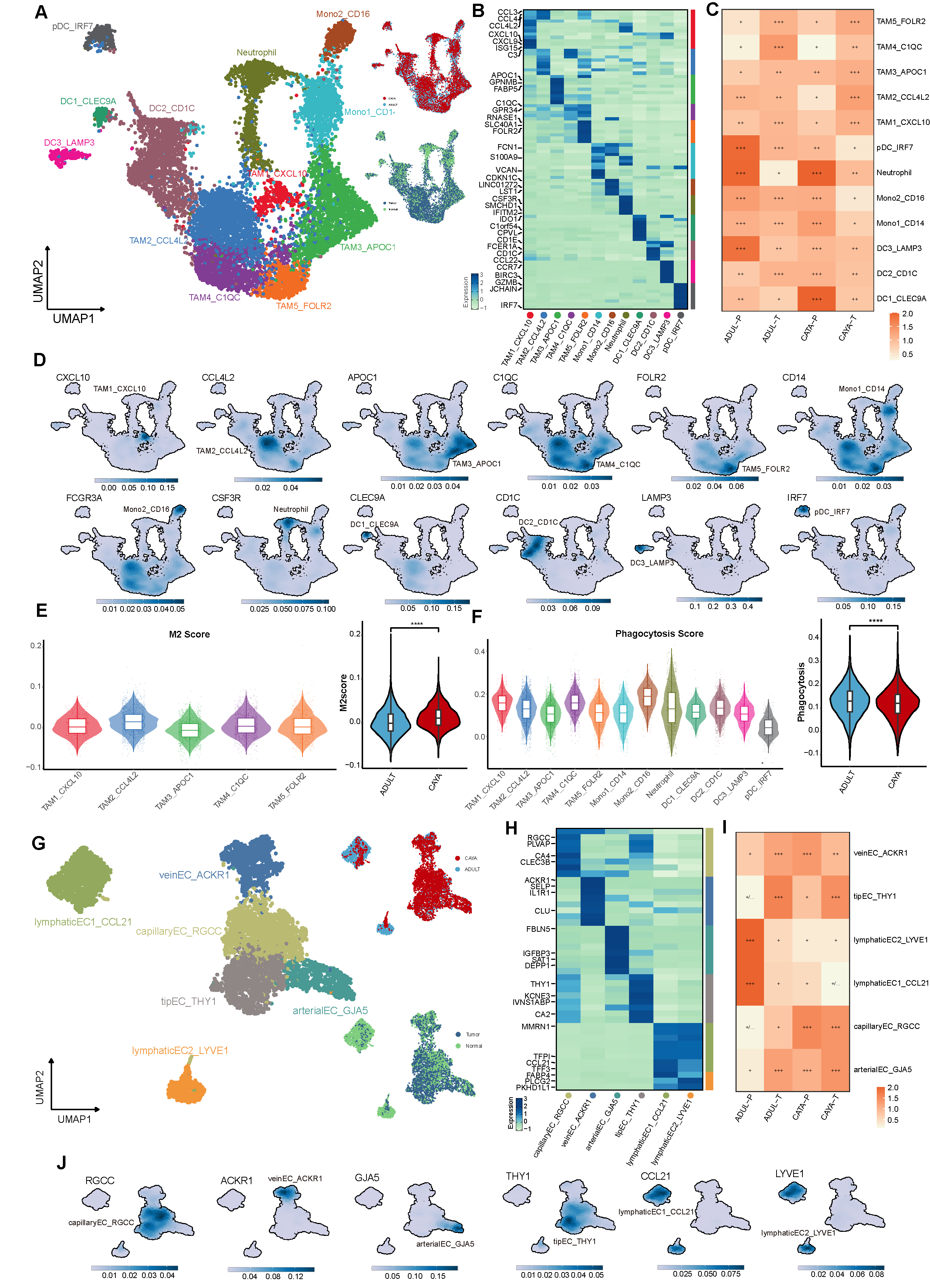

Supplement: Supplementary file 6 — Supplemental Figure 5 [file ADVS-12-e17672-s010.jpg]

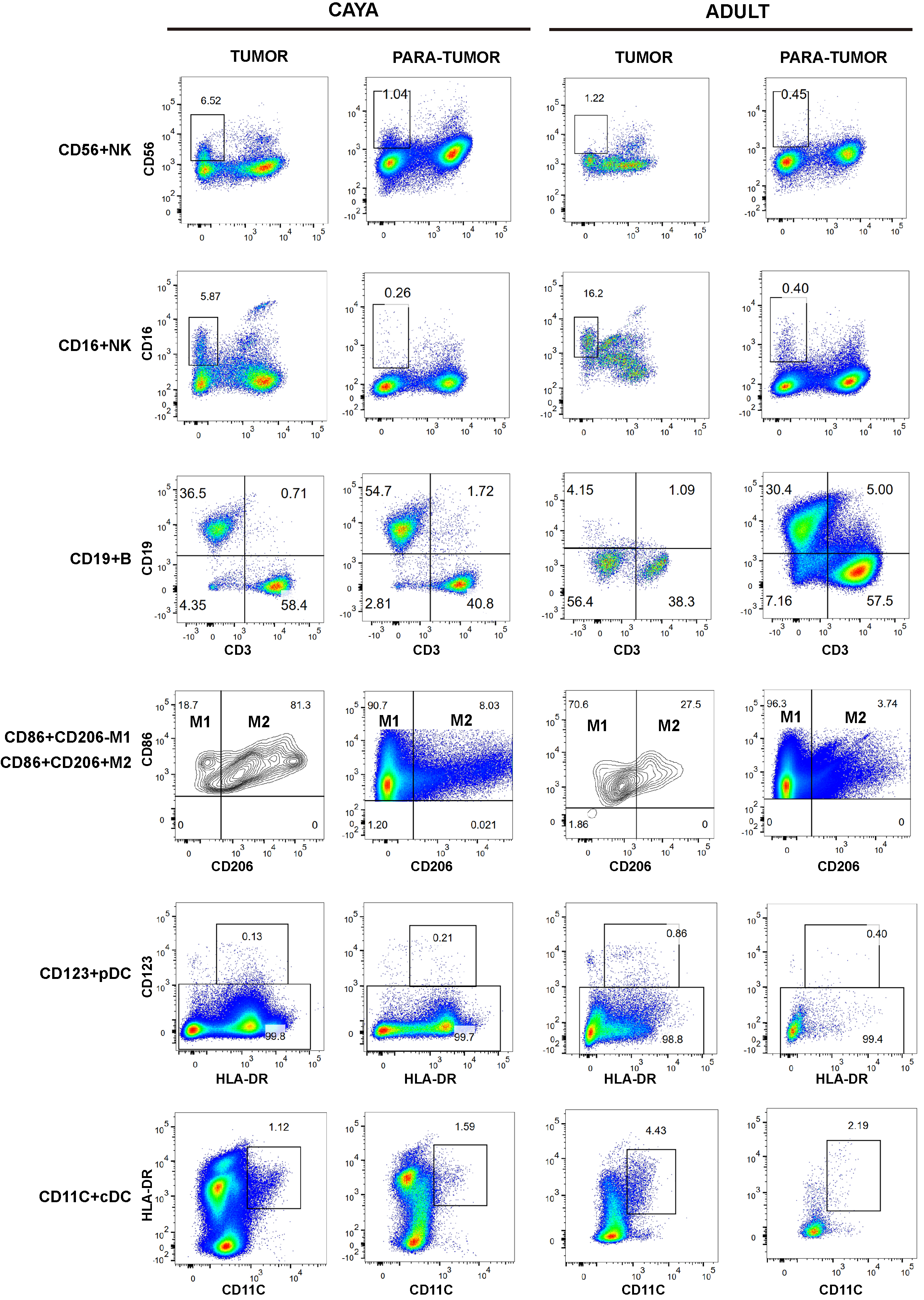

Supplement: Supplementary file 7 — Supplemental Figure 6 [file ADVS-12-e17672-s014.jpg]

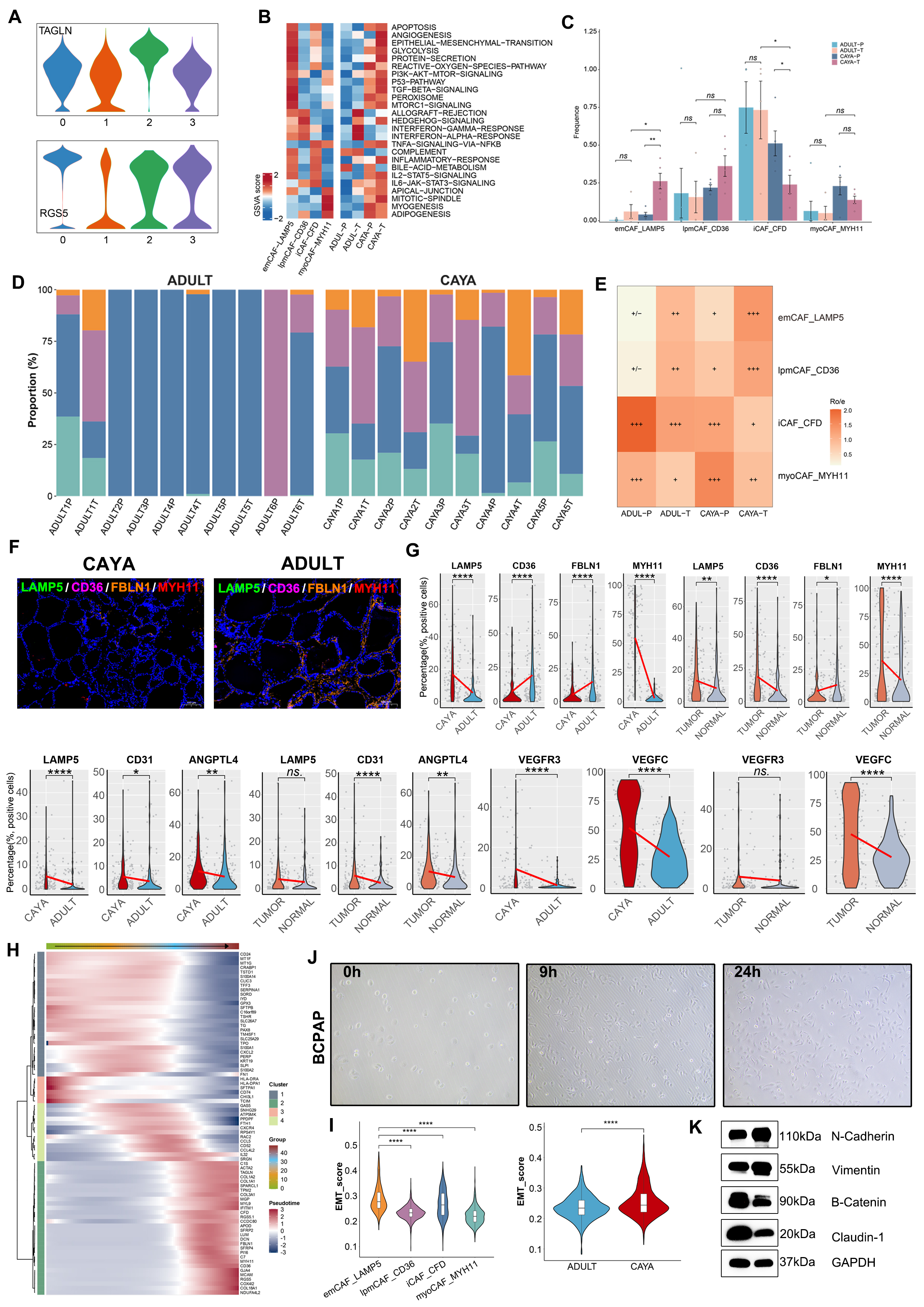

Supplement: Supplementary file 8 — Supplemental Figure 7 [file ADVS-12-e17672-s001.jpg]

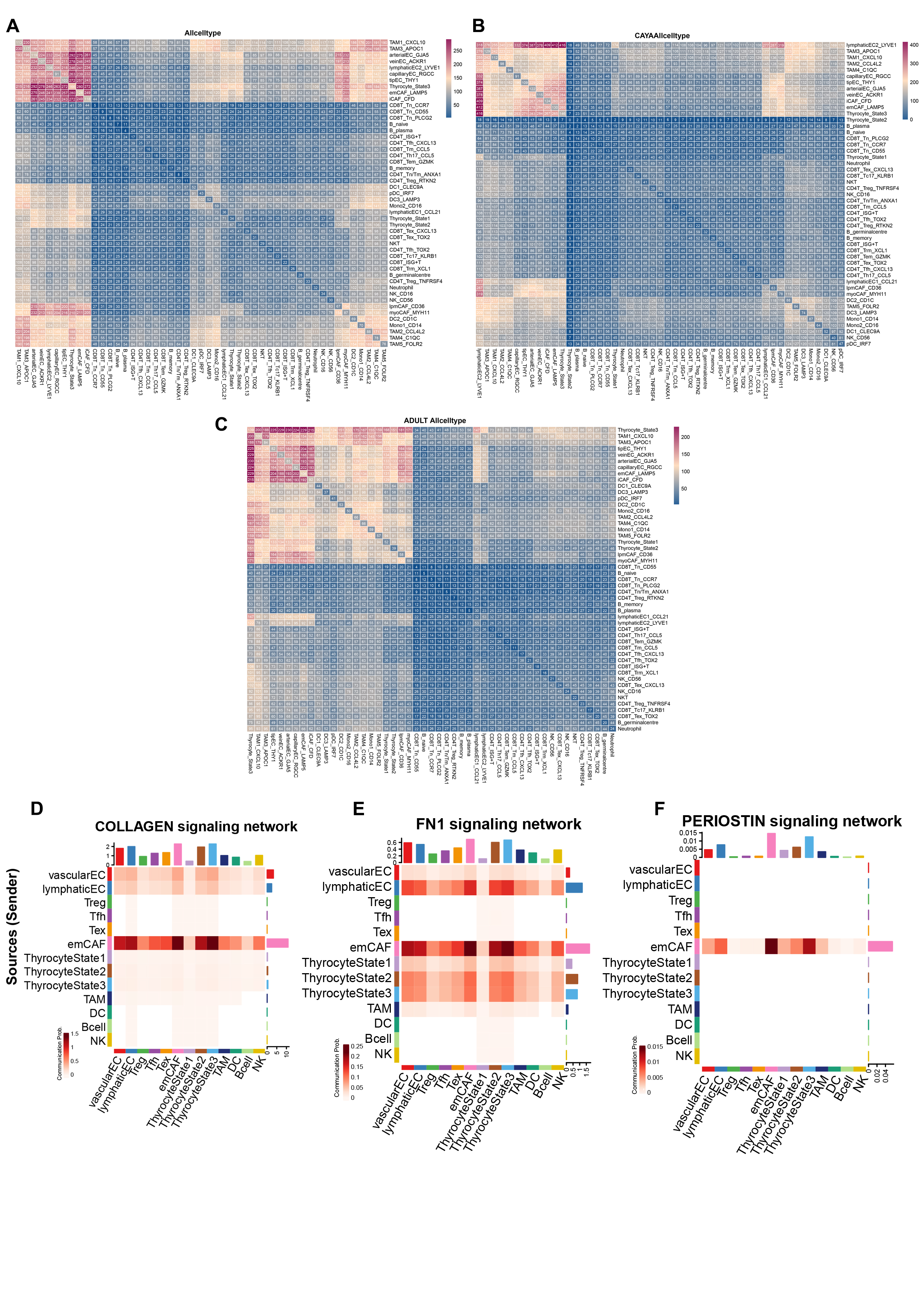

Supplement: Supplementary file 9 — Supplemental Figure 8 [file ADVS-12-e17672-s003.jpg]

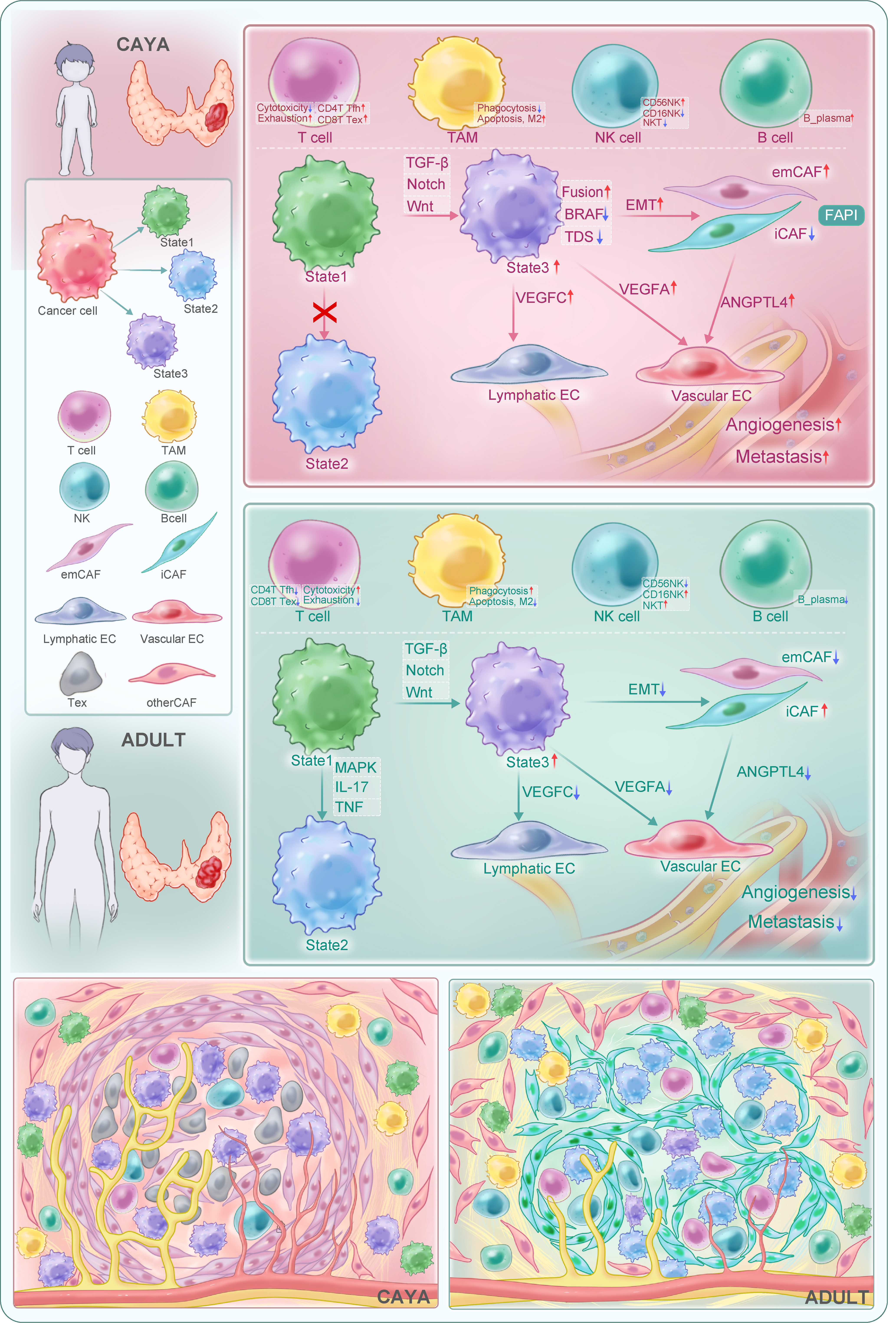

Supplement: Supplementary file 10 — Supplemental Figure 9 [file ADVS-12-e17672-s013.jpg]
