# Supplementary material for: Single‐Cell RNA Sequencing Reveals the Heterogeneity in Differentiation Trajectory and Tumor Microenvironment Leading to More Aggressive Phenotypes of Papillary Thyroid Cancer in Children and Young Adult Patients
Source: Adv Sci (Weinh). 2025 Jul 28;12(39):e17672. doi: 10.1002/advs.202417672 (PMC12533407; doi:10.1002/advs.202417672)
Supplement: Supplementary file 11 — Supporting Tables [file ADVS-12-e17672-s008.docx]

**Table S1. General information on the single cell sequencing samples.**

| Sample | Age | Gender | TNM | Size(cm) | ETE | HT | Subtype | Gene mutation* |
| --- | --- | --- | --- | --- | --- | --- | --- | --- |
| CAYA1 | 14 | Male | T4aN1bM0 | 4.5 | YES | NO | Classical | BRAFV600E |
| CAYA 2 | 11 | Female | T2N0M0 | 2.0 | NO | YES | DS-PTC | CCDC6-RET Fusion |
| CAYA 3 | 16 | Male | T1N1aM0 | 2.0 | NO | NO | Classical | BRAFV600E |
| CAYA 4 | 16 | Female | T2N1bM0 | 2.2 | NO | YES | DS-PTC | CCDC6-RET Fusion |
| CAYA 5 | 9 | Female | T4aN1bM0 | 2.8 | YES | YES | Classical | BRAFV600E |
| ADULT1 | 29 | Female | T2N1bM0 | 3.5 | NO | YES | Classical | BRAFV600E |
| ADULT2 | - | - | T4aN0M0 | 9.5 | YES | YES | Classical | BRAF V600E |
| ADULT3 | - | - | T4aN1bM0 | 2.4 | YES | YES | Classical | BRAF V600E |
| ADULT4 | - | - | T1bN1bM0 | 1.7 | NO | NO | Classical | / |
| ADULT5 | - | - | T4aN1bM0 | 2.7 | YES | YES | Classical | / |
| ADULT6 | - | - | T1bN1aM0 | 1.7 | NO | NO | Classical | BRAF V600E |

Note: ETE: extrathyroidal extension; HT: Hashimoto thyroiditis; DS-PTC: Diffuse Sclerosing variant papillary thyroid carcinoma; Gene mutation: the test was from 8-gene panel of next-generation sequencing.

**Table S2. The Ucell markers used for all cells, CD4+T cells and CD8+T cells.**

| **Figure1E** | **Markers** |
| --- | --- |
| CD4 cells | CD4, CCR7, IL7R, CTLA4, FOXP3 |
| CD8 cells | CD8A, CD8B |
| NK cells | FGFBP2, KLRD1, NKG7, GNLY |
| B cells | MS4A1, CD79A, CD19 |
| Myeloid cells | LYZ, CD68, CD163, CD14, C1QA, C1QB, CD86, VSIG4, S100A9, S100A8, LAMP3, IDO1, IDO2, CD1E, CD1C, LILRA4, IL3RA |
| Endothelial cells | PECAM1, VWF |
| Fibroblasts | TAGLN, RGS5 |
| Thyrocytes | CD24, CLU, TG, EPCAM |
|  |  |
| **Figure 2A** |  |
| CD4T_TnTm_ANXA1 | CCR7, LEF1, GPR183, KLF2, LDHB, PLAC8, CD55, YPEL5, NR4A2, PTGER4, EGR1 |
| CD4T_Tfh_CXCL13 | CXCL13, NMB, ITM2A, IL6ST, CD200, LAG3, PDCD1, NR3C1, TOX, FKBP5, BTLA, TNFSF8, TOX2, IGFL2, GNG4, PTMS, IFITM1, TSHZ2 |
| CD4T_Treg_RTKN2 | RTKN2, FOXP3, IL32, HLA-DRB1, CARD16, PMAIP1, TIGIT, IKZF2, IL2RA, HPGD, CD74, LAYN, SELL, BATF, TBC1D4, STAM, LTB, IL10RA, SAMHD1 |
| CD4T_Th17_CCL5 | ANXA1, CCL5, KLRB1, IL7R, GZMA, GZMK, FKBP11, ERN1, MGAT4A, VIM, HOPX, TPT1, DPP4, IFNGR1, MYBL1, CD40LG, FLT3LG, RORA, CCR6, ANKRD28, CAPG, CEBPD, CCL20 |
| CD4T_Treg_TNFRSF4 | TNFRSF4, TNFRSF18, FOXP3, IL32, IL2RA, BATF, TNFRSF9, LAIR2, CD74, PMAIP1, CTLA4, LAYN, IL1R2, TIGIT, EBI3, IL2RB, NAMPT, TBC1D4, LTA, HLA-DRB1 |
| CD4T_Tfh_TOX2 | TOX2, GNG4, ITM2A, PDCD1, NR3C1, IL6ST, TOX, CXCL13, TNFSF8, CD200, FKBP5, TCF7 |
| CD4T_ISG_T | MX1, IFI44L, IFIT3, IFIT1, RSAD2, USP18, CMPK2, HERC5, IFI6, ISG15, MX2, IFIT2, IRF7, STAT1, PLSCR1, TNFSF10, SP100, IFI16 |
| **Figure S3A** |  |
| CD8T_Tn_CCR7 | LEF1, TCF7, KLF2, LTB, CCR7, IL7R, SELL, MAL, EEF1A1, TRABD2A, TPT1, EEF1B2, NOSIP, PABPC1 |
| CD8T_Tem_GZMK | EOMES, GZMK, CD74, DUSP2, CMC1, CST7, SH2D1A, CRTAM, GZMA, CCL5, IL32, CCL4, HLA-DRB1, HLA-DPA1, COTL1, HLA-DPB1, HLA-DQA1, HLA-DQB1, HLA-DRB5, ITM2C, APOBEC3G, GZMH, GZMM, NKG7, PLEK |
| CD8T_Tm_CCL5 | ZFP36L2, ZFP36, ANXA1, LMNA, FTH1, RGCC, S100A4 |
| CD8T_Trm_XCL1 | ZNF683, HOPX, ID2, ZFP36L2, CKLF, IL32, XCL1, CCL5, XCL2, CXCR3, CAPG, S100A4, LGALS1, ITGA1, LDLRAD4 |
| CD8T_Tex_CXCL13 | TOX, CXCL13, GZMA, CCL3, CCL5, CCL4, IFNG, CD74, CXCR6, HAVCR2, CD27, LYST, PDCD1, DUSP4, CTLA4, TNFRSF9, HLA-DQA1, HLA-DRB1, RBPJ, FAM3C, GZMB, KRT86, TIGIT, PHLDA1 |
| CD8T_Tn_CD55 | LEF1, TCF7, KLF2, TXK, BACH2, CCR7, IL7R, SELL, EEF1A1, ACTN1, TRABD2A, EEF1B2, NELL2, NOSIP, PABPC1 |
| CD8T_Tn_PLCG2 | LEF1, TCF7, TXK, LTB, CCR7, IL7R, SELL, MAL, ACTN1, TRABD2A, EEF1B2, NOSIP |
| CD8T_Tc17_KLRB1 | ZBTB16, CEBPD, RORA, TNF, CCR6, IL7R, IL18RAP, KLRB1, NCR3 |
| CD8T_Tex_TOX2 | TOX, PRDM1, GZMK, CXCL13, CD74, CD27, PDCD1, DUSP4, CTLA4, TOX2, GEM, PDCD1, GNG4, TNFRSF4, NMB |
| CD8T_ISG_T | PLSCR1, STAT1, IRF7, SP100, TNFSF10, IFIT1, RSAD2, IFIT3, IFI44L, MX1, IFI6, OAS1, CMPK2, ISG15, OAS3 |

**Table S3. The statistic results of flow cytometry.**

| **[(Average±SD) %]** | **CAYA** | | **ADULT** | |
| --- | --- | --- | --- | --- |
|  | **Tumor** | **Paratumor** | **Tumor** | **Paratumor** |
| CD3+CD4+T | 23.8±5.2*▲ | 39.5±5.5▲ | 27.9±6.7* | 45.2±9.9 |
| CD25+CD127-Treg | 14.5±3.9*▲ | 3.3±1.1▲ | 9.8±3.5*▲ | 3.7±2.1▲ |
| CD3+CD8+T | 13.8±3.7* | 15.0±4.6 | 22.4±5.1* | 16.4±6.1 |
| CD56+ CD3-NK | 5.4±2.7*▲ | 0.8±0.3▲ | 0.9±0.6* | 0.2±0.4 |
| CD16+ CD3- NK | 4.3±1.6*▲ | 0.3±0.2▲ | 13.5±4.1*▲ | 0.5±0.2▲ |
| CD86+CD206-M1 | 29.4±13.4*▲ | 81.2±15.7▲ | 58.7±21.8*▲ | 89.1±8.5▲ |
| CD206+M2 | 64.9±17.9*▲ | 9.9±7.4▲ | 35.4±11.2*▲ | 8.7±6.1▲ |
| CD123+HLA-DR+pDC | 0.23±0.15 | 0.16±0.11 | 0.62±0.33 | 0.28±0.25 |
| CD11C+HLA-DR+cDC | 0.81±0.37* | 1.43±0.44 | 3.96±1.41* | 1.97±0.42 |
| CD3-CD19+B | 28.3±9.8* | 35.1±17.4 | 11.4±6.7* | 31.9±14.3 |

Note: # represents the comparison of cell proportions in peripheral blood between CAYA (n=10) and adult (n=10) PTC patients, with *P* < 0.05; * represents the comparison of cell proportions in tumor tissues between CAYA and adult PTC patients, with *P* < 0.05; ▲ represents the comparison of tumor to paratumor cell proportions in either CAYA or adults' tissues, with *P* < 0.05. *P* values were determined by Mann-Whitney U test.

**Table S4. The quantitative statistics for mxIHC panels.**

| **LAMP5/CD31/ANGPTL4/PAN-CK (related to Figure 7G)** | | | | | | |
| --- | --- | --- | --- | --- | --- | --- |
| **Percentage of Positive Cell (%)** | | | | | | |
| **Markers** | **ADULT (Mean±SE)** | **CAYA (Mean±SE)** | ***P* value** | **Normal (Mean±SE)** | **Tumor (Mean±SE)** | ***P* value** |
| **LAMP5** | 1.964±0.393 | 5.372±0.543 | 0.000 | 2.903±0.474 | 4.152±0.490 | 0.069 |
| **CD31** | 3.354±0.574 | 5.314±0.541 | 0.014 | 2.582±0.334 | 5.787±0.675 | 0.000 |
| **ANGPTL4** | 7.856±0.869 | 11.278±0.923 | 0.007 | 7.533±0.924 | 11.187±0.865 | 0.004 |
| **Density of Positive Cell** | | | | | | |
| **LAMP5** | 111.513±22.716 | 272.869±31.748 | 0.000 | 133.604±27.294 | 235.160±27.757 | 0.010 |
| **CD31** | 202.595±35.520 | 293.308±33.059 | 0.065 | 131.445±21.996 | 346.938±40.431 | 0.000 |
| **ANGPTL4** | 386.081±65.403 | 602.434±58.349 | 0.015 | 371.102±75.666 | 591.807±49.281 | 0.013 |
| **Intensity of Positive Cell** | | | | | | |
| **LAMP5** | 9.350±0.434 | 11.069±0.213 | 0.001 | 9.133±0.442 | 11.072±0.259 | 0.000 |
| **CD31** | 15.194±0.366 | 16.054±0.380 | 0.105 | 14.330±0.345 | 16.731±0.372 | 0.000 |
| **ANGPTL4** | 19.477±0.455 | 22.556±0.374 | 0.000 | 19.316±0.460 | 22.358±0.388 | 0.000 |
|  | | | | | | |
| **LAMP5/CD36/FBLN1/SMMHC/COL1A (related to Figure 5D)** | | | | | | |
| **Percentage of Positive Cell (%)** | | | | | | |
| **Markers** | **ADULT (Mean±SE)** | **CAYA (Mean±SE)** | ***P* value** | **Normal (Mean±SE)** | **Tumor (Mean±SE)** | ***P* value** |
| **LAMP5** | 5.421±0.768 | 16.869±1.308 | 0.000 | 8.327±1.110 | 13.248±1.160 | 0.003 |
| **CD36** | 19.431±1.655 | 7.444±1.048 | 0.000 | 7.944±1.090 | 18.673±1.633 | 0.000 |
| **FBLN1** | 15.125±1.676 | 5.164±0.665 | 0.000 | 12.904±1.593 | 8.043±1.137 | 0.012 |
| **SMMHC** | 3.036±0.286 | 54.836±2.640 | 0.000 | 18.831±2.254 | 36.121±3.056 | 0.000 |
| **Density of Positive Cell** | | | | | | |
| **LAMP5** | 268.860±41.612 | 914.335±75.094 | 0.000 | 382.837±58.391 | 754.100±67.163 | 0.000 |
| **CD36** | 1025.535±107.332 | 448.417±67.755 | 0.000 | 294.479±39.052 | 1145.835±110.860 | 0.000 |
| **FBLN1** | 703.911±89.433 | 250.609±34.992 | 0.000 | 631.065±86.298 | 356.799±56.763 | 0.007 |
| **SMMHC** | 139.516±13.390 | 3161.005±210.64 | 0.000 | 751.942±99.115 | 2341.446±223.042 | 0.000 |
| **Intensity of Positive Cell** | | | | | | |
| **LAMP5** | 8.063±0.216 | 8.180±0.145 | 0.657 | 7.678±0.234 | 8.508±0.129 | 0.001 |
| **CD36** | 26.887±0.367 | 22.011±0.533 | 0.000 | 22.255±0.605 | 26.543±0.307 | 0.000 |
| **FBLN1** | 18.091±0.338 | 15.528±0.282 | 0.000 | 17.569±0.338 | 16.226±0.316 | 0.004 |
| **SMMHC** | 8.231±0.096 | 13.280±0.389 | 0.000 | 10.398±0.351 | 10.904±0.342 | 0.304 |
|  | | | | | | |
| **PAN-CK/VEGFR3/VEGFC (related to Figure 7H)** | | | | | | |
| **Markers** | **ADULT (Mean±SE)** | **CAYA (Mean±SE)** | ***P* value** | **Normal (Mean±SE)** | **Tumor (Mean±SE)** | ***P* value** |
| **Percentage of Positive Cell (%)** | | | | | | |
| **VEGFR3** | 1.235±0.187 | 9.281±1.178 | 0.000 | 3.774±0.749 | 5.752±0.877 | 0.090 |
| **VEGFC** | 26.884±1.390 | 51.789±2.642 | 0.000 | 27.414±1.514 | 47.688±2.455 | 0.000 |
| **Density of Positive Cell** | | | | | | |
| **VEGFR3** | 61.859±10.297 | 596.415±90.492 | 0.000 | 3.774±0.749 | 5.752±0.877 | 0.090 |
| **VEGFC** | 1290.313±77.466 | 3121.834±222.934 | 0.000 | 27.414±1.514 | 47.688±2.455 | 0.000 |
| **Intensity of Positive Cell** | | | | | | |
| **VEGFR3** | 9.235±0.263 | 11.830±0.198 | 0.000 | 10.598±0.308 | 10.195±0.220 | 0.283 |
| **VEGFC** | 20.566±0.240 | 20.694±0.710 | 0.852 | 18.067±0.359 | 23.001±0.492 | 0.000 |

Note: The comparison of percentage, density and intensity of positive cell between CAYA (n=78) and adult (n=80) PTC patients, normal (n=143) and tumor (n=158) tissues. *P* values were determined by two-side Student’s t-test.
